# The adaptation chip: repurposing the principles of the ichip for guiding in situ experimental evolution

**DOI:** 10.1093/ismeco/ycag053

**Published:** 2026-04-03

**Authors:** Laura M Kaminsky, Javad Sadeghi, Emily Grandinette, Terrence H Bell

**Affiliations:** Department of Plant Pathology and Environmental Microbiology, The Pennsylvania State University, University Park, PA 16802, United States; Boyce Thompson Institute, Ithaca, NY 14853, United States; Department of Physical and Environmental Sciences, University of Toronto – Scarborough, Toronto, ON, M1C 1A4, Canada; Department of Plant Pathology and Environmental Microbiology, The Pennsylvania State University, University Park, PA 16802, United States; Department of Plant Pathology and Environmental Microbiology, The Pennsylvania State University, University Park, PA 16802, United States; Department of Physical and Environmental Sciences, University of Toronto – Scarborough, Toronto, ON, M1C 1A4, Canada

**Keywords:** experimental evolution, adaptive evolution, in situ, soil microbiology

## Abstract

Soil microbial ecosystems are complex and difficult to replicate in laboratory settings. It is often unclear which pressures most strongly shape microbial survival and evolution in situ, and new methods are needed to intersect the manipulative power of the lab with the reality of field environments. One recent innovation was the “isolation chip,” in which many new microbial isolates could be cultured on agar within a buried diffusion chamber while exposed to environmental inputs through fine-pored membranes. Here, we created a modified version of this device containing biologically-cleared soil instead of agar, to trial an in situ reverse ecology experimental evolution approach. Using these “adaptation chips (aChips)” we exposed populations of two different soil-dwelling bacteria (*Priestia megaterium* and *Streptomyces lydicus*) to several farm soils in the Northeast US for up to two years, documenting mutations arising in the evolving populations. While evolution was remarkably slow in the field, *P. megaterium* populations accumulated many mutations pre-burial during aChip construction which seemingly reflected zinc limitation in the aChip carrier soil. Although post-burial mutations were observed in both *P. megaterium* and *S. lydicus* populations, they remained at low frequency and did not display parallelism between aChips buried at the same sites, indicating a lack of strong positive selection and/or limited generations of population growth within the aChip. We suggest several improvements to aChip design to facilitate greater evolutionary progression, including a larger within-aChip soil volume and fewer cells initially secured inside the aChip.

## Introduction

Soil environments are an important habitat for many plant-beneficial microbes [1–3]. There is widespread interest in the practice of inoculating such microbes into agricultural soils, but microbial products have proven to be unreliable [4–7]. One common explanation is poor in-soil survival of inoculated microbes [8]. When introduced to an unfamiliar soil, microbes face many stressors both biotic (e.g. competition with, predation by, or direct antagonism from native microbiota [9–11]) and abiotic (e.g. suboptimal soil pH, moisture, nutrient availability, or texture/structure [7]). Soils are remarkably heterogenous at small spatial scales [12], and the unique ecological and environmental conditions present in any given soil may not be well described. An improved understanding of the most influential biotic and abiotic stressors in different soils could therefore help us identify constraints on microbial inoculant establishment.

To this end, tracking microbial evolution in situ could be a useful tool. In a typical microbial evolution study, an initially isogenic population is incubated for many generations under experimentally imposed conditions. Genetic changes that sweep to high frequency are tracked, revealing genomic targets of selection at the molecular level [13–17]. In vitro studies of this nature have yielded key insights into microbial evolution in the face of single stressors, including those that a microbe might encounter in soil (e.g. competition for resources [18, 19], predation [20, 21], suboptimal pH [22], and nutrient limitation [23]). However, it is difficult to predict which selective pressure may be the most important in a soil or how they act in combination. To this end, experimental evolution conducted in situ in diverse soil environments could generate genetic signatures that reveal the most influential ecological forces at each site—the so-called reverse ecology approach [24]. Yet few such experiments are performed, especially in non-sterile soils, as it can be challenging to contain, recapture, and ensure the survival of the focal evolving species [14].

To overcome these challenges, we have repurposed the design of the isolation chip or “ichip” recently developed by Berdy, Spoering [25]. An ichip consists of an array of small, individual chambers, each containing agar, onto which microbial cells from environmental samples (e.g. diluted soil slurry) are spread. The ichip is then sealed with a fine membrane (~30 nm pore size) and buried in the environment of origin, allowing contained microbes to grow while still receiving relevant inputs from their native environment (soil compounds, extracellular metabolites of other microbes, and possibly phage). Here, we trial the use of modified ichips, which we refer to as adaptation chips or “aChips,” to conduct in situ experimental evolution on two species of soil-dwelling bacteria: *Priestia megaterium* (formerly *Bacillus megaterium*) [26], a phosphorus-solubilizer, and *Streptomyces lydicus*, a biocontrol against pathogenic fungi and bacteria. Our aChips are filled with an autoclaved soil rather than agar, to more closely approximate the structural properties of soil, and are inoculated with a single known isolate rather than trying to capture unknown taxa from environmental samples. The principle of environmental exposure remains the same.

**Figure 1:**
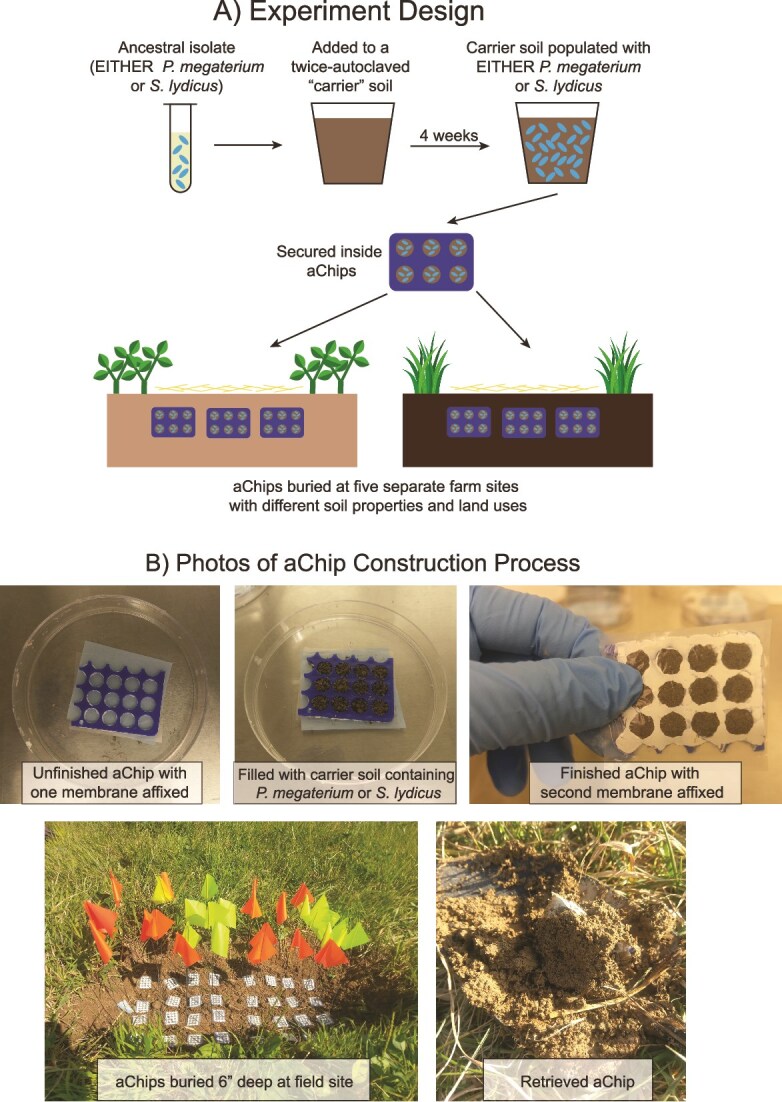
(A) Schematic of the experimental process. (B) Construction, deployment, and retrieval of adaptation chips or “aChips” in a soil environment.

We buried our aChips at five farm sites across Pennsylvania and New York, USA for up to two years (Fig. 1A). Periodically, we conducted shotgun metagenomic sequencing on *P. megaterium* and *S. lydicus* populations recovered from the aChips to track mutations that arose over time. We demonstrate that these focal populations survived inside the aChips, but that evolutionary change after aChip burial was very limited. This indicates that microbial growth and evolution in soil may proceed remarkably slowly.

## Materials and Methods

### Bacterial isolates

The ancestral isolates of *Priestia megaterium* and *S. lydicus* were obtained from commercial products: MegaPhos (Blacksmith BioScience Inc., Spring, TX, USA) and Actinovate (Novonesis, Lyngby, Denmark), respectively. Both products are approved for use on certified organic farms. The product powders were suspended in sterile water then plated on tryptic soy agar (TSA) or International Streptomyces Project medium 4 (ISP4) [27], respectively. The resulting colonies were purified and confirmed as *P. megaterium* or *S. lydicus* with whole genome sequencing. These genomes were used as the reference for all subsequent mutation analysis (Fig. S1).

### aChip construction and burial

Construction of aChips followed Berdy, Spoering [25], with some modifications (Fig. 1B). For details, see the Supplemental Information. To fill the aChips, we used a twice-autoclaved “carrier soil” that had been inoculated with either the ancestral isolate of *P. megaterium* or *S. lydicus* and incubated at 20°C and 85% relative humidity for four weeks. We conducted this pre-burial incubation to prime our microbes to live in a simple soil environment after spending time in laboratory conditions. The abiotic properties of the autoclaved carrier soil are displayed in Table S1. We chose to use one common carrier soil for every aChip so that this priming would be consistent across the experiment, and so that we would know that differential between-site evolution was due to field conditions, not carrier soil conditions. Additionally, we wished to establish a large initial population of our focal species inside the aChips before deployment, reasoning that this would provide a larger pool on which selective forces could act. At the time of construction, several samples of the carrier soil were collected for DNA extraction and metagenomic sequencing.

Sixteen replicate aChips per species were buried in late August 2019 at each of five farm sites (Site IDs: BC, BT, CCC, GH, and PVF) with varying land uses across Pennsylvania and New York (Table S1). The burial site soils varied in abiotic properties (Table S1), with pHs ranging from 5.62 to 7.12, organic matter content ranging from 2.2% to 4.87%, and unique combinations of nutrient availabilities. Additionally, the resident microbes as determined by 16S rRNA gene sequencing varied by site at the time of burial (Fig. S2), though we note that these taxonomic compositions may have changed over the course of the experiment. Each aChip was buried ~3 cm from adjacent aChips at a soil depth of 6 inches (Fig. 1B). The burial area was covered with a thick layer of straw.

### aChip retrieval and sampling

From each farm site, aChips were retrieved from the field after 3, 10, and 24 months. To recover the carrier soil, a sterilized razor was used to cut away the membrane around each well, and well contents were deposited into a sterile 2-ml microcentrifuge tube. All carrier soil from a single aChip was put into the same tube, excluding any wells that were visibly compromised by tears to the membrane.

**Figure 2:**
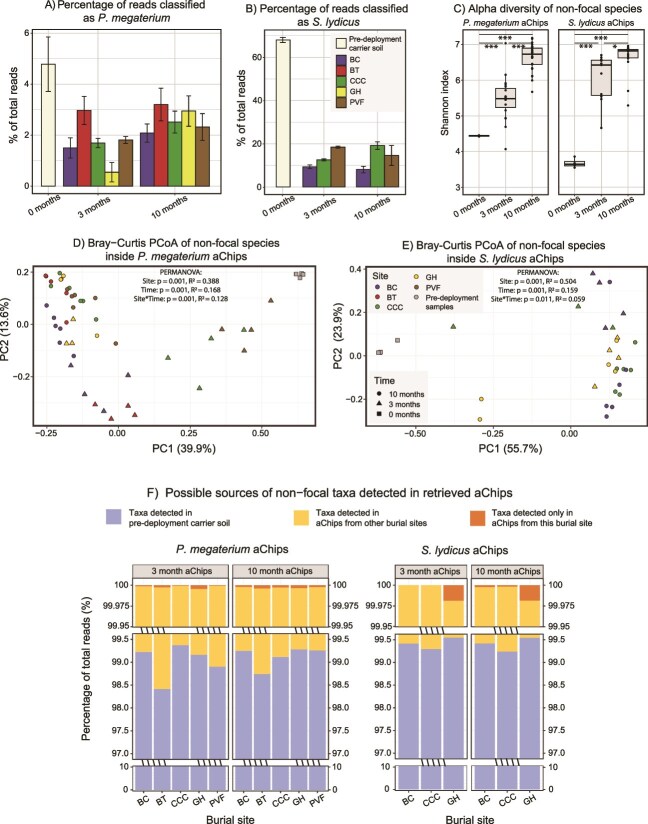
Non-focal species present inside aChip carrier soil. See also Fig. S4 and S5. (A) Percentage of shotgun metagenomic reads from aChip carrier soil classified by kraken as originating from *P. megaterium* in *P. megaterium*-inoculated aChips. (B) Percentage of shotgun metagenomic reads from aChip carrier soil classified by kraken as originating from *S. lydicus* in *S. lydicus*-inoculated aChips. (C) Alpha diversity (Shannon) of non-focal species within the aChips across time. Asterisks denote Tukey’s HSD test *P*-value results following ANOVA testing the effect of time (^***^ < .001, ^**^ < .01, ^*^ < .05). PCoA of Bray–Curtis distances in non-focal species community composition within (D) *P. megaterium* aChips and (E) *S. lydicus* aChips. Point shapes indicate sampling time while point colors indicate burial site. (F) Possible sources of non-focal taxa detected in retrieved aChips. Of all the shotgun reads detected in a set of aChips, we tallied what percentage were detected in the pre-deployment carrier soil, in aChips from one of the other burial sites, or exclusively within aChips from a single burial site. Note the breaks and changes in scale in the y-axis, to better display the latter two categories of taxa.

We initially planned to conduct mutational analyses of the evolving focal populations via shotgun metagenomic sequencing on DNA extracted from the carrier soil, with the assumption that these DNA extracts would be dominated by *P. megaterium* and *S. lydicus* reads. However, this assumption proved untrue for our 3-month and 10-month samples, and for our pre-deployment *P. megaterium* carrier soil (Fig. 2A and B and Fig. S3). Using these soil metagenomic reads for mutation analysis would have resulted in suboptimal coverage of the focal ancestral isolate genomes once we filtered out contaminating species (Fig. S3A). We instead used this data to examine non-focal species diversity within the aChips over time, but omitted this analysis on the 24-month samples due to sequencing cost.

For our mutation analysis, we instead collected a pool of 100 distinct isolates recaptured from each aChip (see Supplemental Information). Similar isolate-pooling approaches have been used before to characterize evolving communities [28–30], but we acknowledge that this approach is liable to miss rare genetic variants (e.g. <1% frequency). Note that 100-isolate pools were also checked for purity, and some *S. lydicus* samples were found to be lacking and therefore excluded from analysis (see Supplemental Information for details).

The number and type of samples collected across the study is listed in Table S2. Difficulties in re-locating aChips or excessively compromised aChips resulted in some differences in replicate numbers across sites and time points. At the final 24-month time point, we opted to focus on just three farm sites due to sequencing costs. For details on DNA extraction and sequencing, please see the Supplemental Information.

### Sequence availability

Raw sequences are available on the NCBI SRA database under BioProject accessions PRJNA933577, PRJNA932007, and PRJNA936108.

### Bioinformatic analysis

Bioinformatic pipelines and analysis goals differed across sample types and are outlined in Fig. S1. See Supplemental Information for details.

### Statistics and data analysis

All figures were generated in R v4.2.1, except Venn diagrams which were generated at the following site: http://bioinformatics.psb.ugent.be/webtools/Venn/. We focused our analysis and discussion on non-synonymous mutations arising within the populations, as have other studies [31], as these have the most straightforward link to selection. Further details on our statistical analysis can be found in the Supplemental Information.

## Results

### Focal and non-focal species populations inside the aChips

We recovered 100+ isolates of the focal species from almost every retrieved aChip, demonstrating aChip utility for containing experimental microbes for at least two years. Using CFU counts from aChip carrier soil dilutions, we estimate that the within-aChip population density of the focal species declined slightly from burial to the final retrieval (2.76 ^*^ 10^7^ vs. 2.20 ± 0.32 ^*^ 10^7^ CFU g^−1^ fresh soil at burial vs. 24 months for *P. megaterium*; 1.02 ^*^ 10^8^ vs. 7.02 ± 0.92 ^*^ 10^7^ CFU g^−1^ fresh soil at burial vs. 24 months for *S. lydicus.* Values listed are mean ± SE). While this does not exclude the possibility of dynamic population fluctuations throughout the experiment, it may also be that the focal species did not undergo much growth or activity while buried. Both *P. megaterium* and *S. lydicus* can form spores and could have gone dormant for long stretches of time.

We also observed other taxa present inside the aChip from the time of construction, whose relative abundance and alpha diversity increased over time (Figs 2A–C). There are three possible sources for these non-focal species: (i) Organisms native to the carrier soil, which survived repeated rounds of autoclaving and grew alongside the focal species; (ii) Organisms introduced during aChip construction; and (iii) Organisms which entered the aChips from the surrounding soil after burial. Upon examining the non-focal taxa detected in the harvested aChips, we determined that >98% of these reads came from taxa also detected in the pre-deployment carrier soil (Fig. 2F). This indicates that almost none of the non-focal species came from the aChip burial sites, confirming that the aChip membranes were an effective barrier against outside microbes. However, the beta diversity of non-focal taxa inside the aChips varied significantly depending on burial site and harvest time (Fig. 2D and E, Fig. S4, Fig. S5), suggesting that the pre-deployment community responded differently to each farm. That is, we could detect some influence of the external environment on the interior contents of the aChips.

Because part of our goal was to expose our focal species to selective forces imposed by other microorganisms, we did not consider the presence of non-focal species inside the aChips to interfere with the purpose of the experiment. But it is worth noting that the list of selective forces at operation in and around the aChips included the biotic contaminants and abiotic conditions of the carrier soil, rather than only the biotic residents and abiotic conditions of the burial sites.

### Mutations arising in *P. megaterium* populations before aChip burial

**Figure 3:**
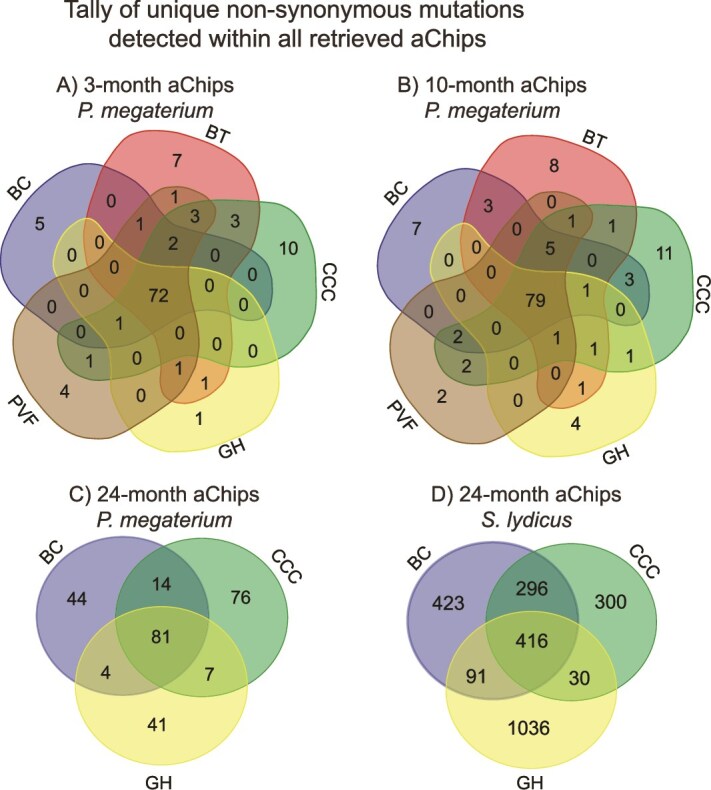
Venn diagrams comparing the non-synonymous mutations detected in *P. megaterium* 100-isolate pools retrieved from each farm after (A) 3 months, (B) 10 months, and (C) 24 months, and D) in *S. lydicus* 100-isolate pools after 24 months. If a mutation appeared in more than one aChip at a given site, it was only counted once in these diagrams. Note that 24-month samples were not sequenced for sites BT and PVF, and that mutation data from 3- and 10-month *S. lydicus* samples were too low coverage to be considered reliable (see Fig. S3).

**Figure 4:**
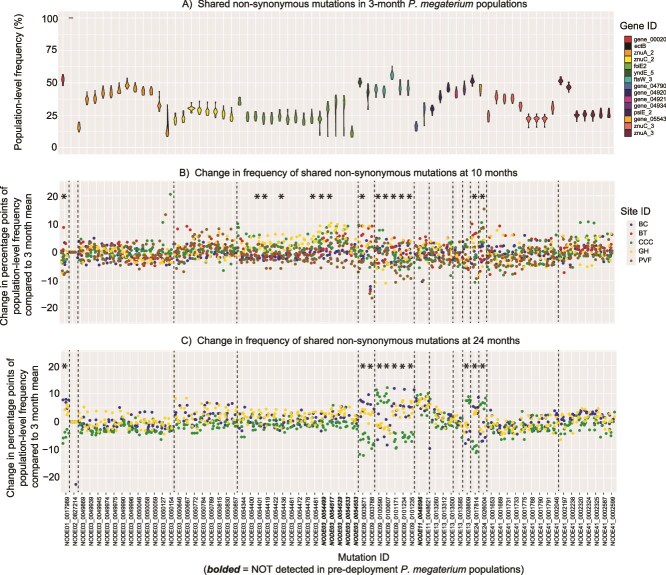
Population-level frequencies of the 69 shared non-synonymous mutations in *P. megaterium* 100-isolate pools across time. Almost all of these mutations were detected in pre-deployment aChip carrier soil, but the few that were not are bolded on the x-axis. X-axis labels correspond to all three panels. (A) Violin plots showing the range in frequency for each individual shared mutation at the 3-month sampling point. The color of the violins corresponds to the gene in which that mutation occurred. (B) Change in frequency of each shared mutation compared to the 3-month mean after 10 months and (C) 24 months. Dashed lines separate the mutation data based on the genes in which they occurred. Each point represents an individual 100-isolate pool and points are colored by aChip burial site. Asterisks denote a significant difference in population-level frequencies in that mutation based on burial site. See Table S4 for two-way ANOVA model results and Tables S5 and S6 for Tukey’s HSD test *P*-values.

When examining the non-synonymous mutations present in the 3- and 10-month *P. megaterium* populations, we found few unique to any given site. Rather, most mutations were detected at every site (Fig. 3A and B). After further analysis, we determined that 69 mutations were detected in nearly every individual aChip harvested throughout the experiment (at least 54 of the 59 total *P. megaterium* aChips sampled). Here forward, we refer to this set of 69 non-synonymous mutations in *P. megaterium* as “shared mutations,” which were housed within 15 unique genes (listed in Table 1). In the 3-month data, most of the shared mutations were detected at 25–50% frequency in the population, with remarkably narrow ranges in frequency across aChips (Fig. 4A). There were no significant differences in any shared mutation frequencies across sites at 3 months (Table S4, Table S5).

**Table 1:** Annotation and functional information for the genes in which the 70 shared non-synonymous mutations occurred. Genes lacking a standard name are instead IDed with the arbitrary number assigned to them during de novo annotation by Prokka. The number of non-synonymous and synonymous mutations detected in these genes and the ratio between them (N:S) is also displayed as mean ± se for all the aChips which possessed mutations in these genes.

**Gene ID**	**Gene annotation**	**General function**	**Average mutation counts**		**N:S**
			**Non-synon.**	**Synon.**	
*gene_00020*	Small acid-soluble spore protein	Sporulation and/or spore germination	1 ± 0	2 ± 0	0.5
*ectB*	Diaminobutyrate—2—oxoglutarate aminotransferase	Biosynthesis of osmoprotectant	1 ± 0	0 ± 0	NA
*znuA_2*	High affinity zinc uptake system binding protein	Nutrient uptake	12.76 ± 0.06	14.07 ± 0.12	0.91
*znuC_2*	High affinity zinc uptake system ATP-binding protein	Nutrient uptake	8 ± 0	21.14 ± 0.06	0.38
*folE2*	GTP cyclohydrase	Biosynthesis of amino acids	15 ± 0	17 ± 0	0.88
*yndE_5*	Spore germination protein	Spore germination	2 ± 0	6.29 ± 0.10	0.32
*ftsW_3*	Putative peptidoglycan transferase	Cell division	5 ± 0	16 ± 0	0.31
*gene_04790*	Hypothetical protein	Unknown	1.93 ± 0.04	3.46 ± 0.07	0.56
*gene_04920*	Hypothetical protein	Unknown	3 ± 0	2 ± 0	1.5
*gene_04921*	Hypothetical protein	Unknown	1 ± 0	2 ± 0	0.5
*gene_04934*	Hypothetical protein	Unknown	1 ± 0	0 ± 0	NA
*psiE_2*	Phosphate starvation induced protein	Phosphate starvation response	1 ± 0	5 ± 0	0.2
*Gene_05543*	IS3 family transposase ISBth8	DNA transposition	1.46 ± 0.06	4 ± 0	0.37
*znuA_3*	High affinity zinc uptake system binding protein	Nutrient uptake	7.62 ± 0.14	18.74 ± 0.15	0.41
*znuC_3*	High affinity zinc uptake system ATP-binding protein	Nutrient uptake	9.16 ± 0.05	27.07 ± 0.10	0.34

Given the ubiquitous occurrence and homogenous frequency of the shared mutations, we suspected that they were present before the aChips were buried. During the lab-based carrier soil incubation, *P. megaterium* populations were likely growing in response to stable and favorable conditions, thereby generating genetic variation. To explore whether the shared mutations were present pre-deployment, we conducted shotgun metagenomic sequencing and mutation analysis on DNA extracted from samples of the carrier soil frozen immediately after aChip construction. We confirmed the presence of all but 6 of the shared mutations (see italicized mutations in Fig. 4), which were perhaps missed due to subpar sequence coverage (Fig. S3A). Taken together, this evidence suggests that the shared mutations had arisen in the 30 days during *P. megaterium* incubation in the sterile carrier soil, before aChip construction.

Of the genes containing the shared mutations (Table 1), several were related to the essential micronutrient zinc. ZnuA and ZnuC are subunits of a high-affinity zinc uptake ABC transporter [32], while FolE2 acts a non-orthologous replacement for the Zn^2+^-dependent enzyme FolE in the biosynthesis of folate (a critical co-factor for many biochemical reactions) under conditions of zinc deficiency [33, 34]. This was noteworthy because the zinc level in the carrier soil was quite low at 1.5 ppm (Table S1), a number generally marked as borderline deficient for crops in Pennsylvania soils [35]. The remaining genes carrying shared mutations had less straightforward links to the properties of the carrier soil. Four genes were annotated as hypothetical proteins, with unknown functions to date. Two genes were related to spore formation and germination: the spore germination protein *yndE* [36] and a small acid-soluble spore protein [37]. The others were *ftsW*, which plays a structural role in cell division [38], *psiE*, which has an unknown function but is upregulated under phosphorus starvation [39], *ectB*, an intermediate enzyme in the biosynthesis of the osmolyte ectoine [40], and an IS3 family transposase [41]. Within only four weeks, the population-level frequencies of many of these mutations rose to >25%. While it is possible that this outcome occurred via purely stochastic processes, some degree of positive selection could have been acting on these mutations to increase their prevalence.

 One final pattern of note was that many of the shared mutations occurring in *ftsW_3*, *folE2*, *znuA_2, znuA_3*, *znuC_2*, and *znuC_3* were found together on single sequencing reads (see Fig. S6 for an example). That is, there appeared to be multiple linked point mutations all occurring *within single isolates* in the 100-isolate pool. There are three possible explanations. First, the mutation analysis program could have consistently misaligned reads when multiple homologous copies of a gene existed within the *P. megaterium* genome. However, we generally found no evidence for misalignment; mutated genes always matched best with their assigned gene copy (Table S7). Second, it is possible that there were horizontal gene transfer (HGT) events to *P. megaterium* from a non-focal species living in the carrier soil, as *P. megaterium* does possess the cellular machinery required for transformation [42]. However, the only support for this idea comes from a relatively low ratio of non-synonymous vs. synonymous mutations within these genes, generally <1:1 (Table 1) in contrast to a typical ratio of 3:1 for newly arisen mutations [43]. Yet the degree of divergence from the ancestral gene is still lower than what we might expect from an HGT event, and there were very few closely related *Priestia* taxa present in the pre-deployment carrier soil (0.027% of total reads). Finally, these genes could simply be hotspots for mutation or especially strong targets for selective pressures in the particular context of lab incubation in the carrier soil.

### Changes in shared mutation frequencies in *P. megaterium* populations after burial

The shared mutations can be viewed as standing genetic variation that existed within the *P. megaterium* populations at the time of aChip burial. Any significant frequency changes in these mutations observed at a particular field site could therefore signal selective forces operating on the population. Note that we used the 3-month mutation frequencies as the baseline for our analysis rather than the pre-deployment frequencies, because we did not consider the latter reliable due to low coverage of the ancestral *P. megaterium* genome (Fig. S3).

Generally, the frequencies of the shared mutations scarcely changed over the course of the 24-month burial. However, after 24 months a subset of the shared mutations had significantly shifted from their 3-month frequencies by up to +/− 10 percentage points (Fig. 4C mutations marked with ^*^, Tables S4, S5, and S6), and the direction of these shifts varied based on burial site. Genes affected by this pattern included a small acid-soluble spore protein, a spore germination protein, a putative peptidoglycan transferase, a hypothetical protein, a phosphate starvation induced protein, and an IS3 family transposase (Tables S4, S5, and S6). Comparatively, shifts in shared mutation frequencies were more subtle at the 10-month time point (Fig. 4B), though there were some statistically significant differences (Fig. 4B, Table S5).

While these site-specific frequency shifts could indicate site-specific selective pressures acting on the *P. megaterium* populations, the magnitude of these changes was still quite small. This provides further evidence that *P. megaterium* evolution in soil environments proceeded very slowly during this experiment. There were significant correlations between burial site abiotic features and the frequencies of certain mutations (Table S8), including a negative association between zinc levels and mutation frequencies in the FolE2 gene, a non-orthologous replacement for a Zn-dependent enzyme (Fig. S7), but this pattern did not hold at 24 months.

### Mutations arising in *P. megaterium* populations after aChip burial

**Figure 5:**
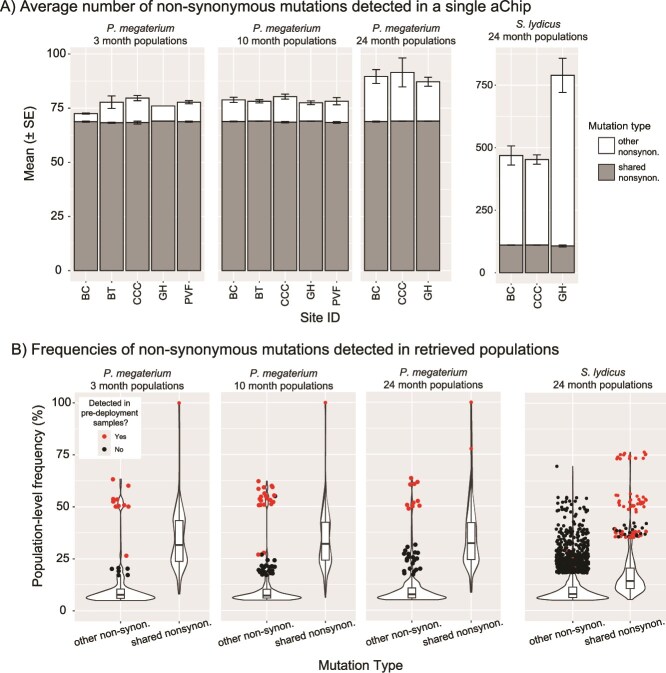
Shared vs. non-shared non-synonymous mutation frequencies in the 100-isolate pools of *P. megaterium* and *S. lydicus*. (A) Average number of non-synonymous mutations detected within a single aChip at each burial site and time point across the experiment. Note the difference in y-axis range for *S. lydicus* populations. (B) Violin plots showing the distribution of frequencies for all shared and non-shared non-synonymous mutations detected in 100-isolate pools across the experiment. Points indicate outlier mutations, and are colored red if they were detected in the corresponding pre-deployment aChip carrier soil.

Very few de novo mutations arose in the *P. megaterium* populations after aChip burial. On average, any particular 100-isolate pool retrieved after 3 months or 10 months carried less than 10 novel non-synonymous mutations (Fig. 5A), and the population-level frequencies of the vast majority of those mutations remained below ~10% (Fig. 5B). The handful of non-shared mutations with higher frequencies (>20%) were almost all detected in pre-deployment populations (red points in Fig. 5B), suggesting that they also arose during carrier soil incubation rather than post-burial. By 24 months, more non-shared non-synonymous mutations were detected per aChip (Fig. 5A). Even so, the population-level frequencies of most mutations not detected pre-deployment remained low (Fig. 5B), and there was no parallelism in genes mutated in replicate aChips from the same site.

### Mutations arising in *S. lydicus* populations

Due to low coverage of the *S. lydicus* genome from the 3-month and 10-month 100-isolate pools (Fig. S3), we unfortunately could not conduct reliable mutation analyses for *S. lydicus* at these two time points. However, in the 24-month samples, it was clear that there were more mutations detected per aChip for *S. lydicus* than for *P. megaterium*, despite similar coverage depth of the ancestral genome for both species (423x ± 18 for *P. megaterium*; 509x ± 29 for *S. lydicus*) and even when accounting for the larger *S. lydicus* genome size (Fig. S8). This implies either that *S. lydicus* is more prone to genetic changes, or that these populations experienced more generations of growth and evolution. Some prior evidence exists for the former explanation. *Streptomyces* genomes are organized as single linear chromosomes, with more conserved central regions containing essential genes and chromosomal arms containing a more variable set of dispensable genes [44]. The chromosomal arms are subject to considerable mutation, such that even closely related taxa can have distinct evolutionary fingerprints [45]. Additionally, some *Streptomyces* taxa can undergo rapid differential evolution within single colonies, leading to distinct mutants at extremely small spatial scales [46]. Either of these factors could have been at work in our *S. lydicus* populations, leading to more rapid genetic change.

**Figure 6:**
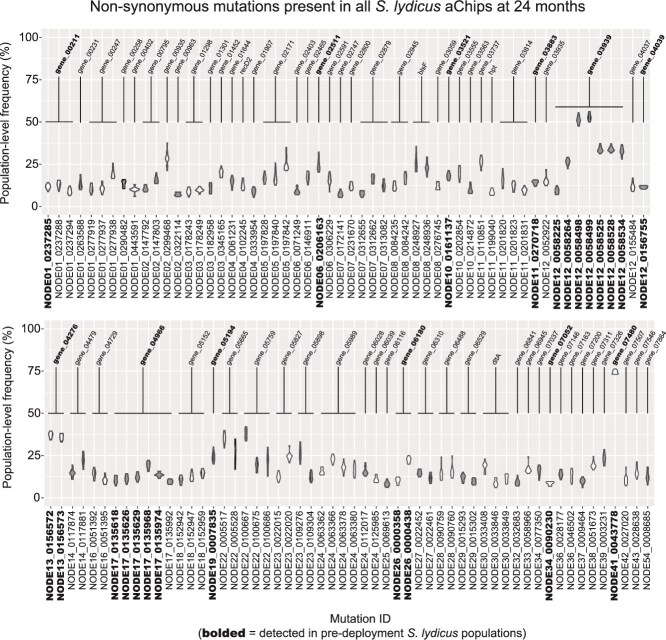
Population-level frequencies of the 112 shared non-synonymous mutations in *S. lydicus* 100-isolate pools at the 24-month sampling point. Some of these mutations were also detected in pre-deployment aChip carrier soil, and are bolded on the x-axis. Violin plots show the range in frequency for each individual shared mutation. The color alternates between white and grey to denote different genes in which mutations occurred. Gene IDs are labelled, and gene annotations can be found in Table S9.

Given the prevalence of pre-deployment mutations for *P. megaterium*, we examined whether there was a similar set of mutations present in *S. lydicus* populations. In general, there was far less overlap across sites in *S. lydicus* (Fig. 3D), but there were 112 mutations that were present in almost every aChip (at least 13 out of 14) at 24 months (Fig. 6). In contrast with the *P. megaterium* shared mutations, these 112 mutations spanned 68 genes (Table S9), with single point mutations in each gene more common than multiple linked mutations concentrated in particular genes. Additionally, the 112 shared mutations generally had frequencies <20% (Fig. 6). We could confirm the presence of only 25 of these mutations in the pre-deployment *S. lydicus* populations (Fig. 6), but this included some of the higher frequency shared mutations. Genes housing >20% frequency, pre-deployment mutations included a TetR family transcriptional regulator, two hypothetical proteins, a permease, an IS1182 family transposase, and an IS5 family transposase (Fig. 6). We also noted that shared mutations made up a much smaller proportion of the total non-synonymous mutations detected in any particular aChip (Fig. 5A). Taken together, there was some limited evidence for the retention of pre-deployment mutations out to 24 months in *S. lydicus* populations, but they were not particularly ubiquitous or dominant.

Excluding the shared mutations, most mutations remained below a 10% frequency in the *S. lydicus* populations (Fig. 5B). Nonetheless, we examined whether there were any genes housing mutations in most of the aChips at one particular site which were never mutated at any other site. Such a pattern of within-site mutation parallelism could reveal evolutionary pressures unique to that site. However, there were no uniquely mutated genes for site CCC and only one uniquely mutated gene for site BC (Table S10). Site GH had a handful of uniquely mutated genes (Table S10), but we note that only three aChips were collected from site GH at 24 months and therefore this list may be overly inclusive. All told, evidence for strong site-specific evolutionary pressures was lacking as the mutations detected in retrieved aChips were largely singular, low frequency, and lacked parallelism.

## Discussion

In this study, we trialed the use of “adaptation chips” for in situ experimental evolution of two soil-dwelling bacteria in five farm soils across Pennsylvania and New York. We conducted metagenomic population re-sequencing after 3, 10, and 24 months of burial to track evolutionary change, with the goal of revealing important ecological selective forces within these environments.

### Limited evolutionary change during aChip burial

Taken all together, our data suggests that the pace of evolution inside the aChips was remarkably slow. We lacked evidence for substantial population growth of the focal species inside the aChips, did not see considerable frequency shifts in the standing genetic variation represented by the shared mutations, and observed few de novo mutations rising to prominence after burial. There was additionally no strong parallelism in mutated genes among replicate aChips buried at the same site.

Given the rapid pace of bacterial evolution in laboratory media [47], the overall lack of evolutionary change upon exposure to field soils was striking. One possible explanation is that our focal species simply went dormant for long stretches after burial. Only ~1 g of soil was contained in each aChip, and the populations may have already reached carrying capacity even before burial. The presence of contaminating taxa inside the aChips may have further limited resources and created competition. Both *P. megaterium* and *S. lydicus* can form spores when they encounter stressful conditions [48, 49], so it is possible that both species sporulated upon or before aChip burial with sporadic germination and population turnover thereafter. More generally, soil is more resource-limited than lab media. A tendency towards dormancy and slow growth is thought to be common and adaptive for many soil-dwelling microorganisms [50–52] given the stressors of life in soil, and may generally impede rapid evolutionary progress.

What we observed may therefore represent de novo mutations as they arose during sporadic spurts of growth rather than mutations that had been the targets of positive selection. However, selection in soil environments may also be a more complex and variable phenomenon, less likely to generate the same evolutionary patterns observed in lab studies. Soil properties are heterogeneous even at small spatial scales [53, 54], such that two aChips buried only centimeters from each other could have been experiencing notably different conditions. For that matter, two different wells in the *same* aChip could be experiencing different selective pressures, or be more subject to genetic drift, and by combining all the wells into one sample we may have obscured finer-scale sweeps. Soil conditions also vary substantially across time. This lack of consistent selective pressures could drive complex within-population dynamics, preventing any single genetic variant from sweeping through the evolving populations.

Another in situ evolution study found similarly weak evidence of positive selection in the field. In Chase, Weihe [55], a *Curtobacterium* isolate was buried for 18 months at five different field sites across a climate gradient, contained inside fine-pored membrane bags filled with a ground grass litter sourced from one of the field sites. Most detected mutations had very low frequencies, and the ratio of non-synonymous to synonymous mutations in the evolving populations did not support the presence of strong selective pressures. One proposed explanation was that the *Curtobacterium* isolate may have already been well adapted to the burial sites, as it was initially isolated from one of them. This stands in contrast to our approach of exposing the focal isolate to completely novel environments, so it is noteworthy that we both found evidence of evolution proceeding slowly. However, Chase et al did observe a handful of parallel, site-specific mutations, a pattern we failed to see. *Curtobacterium* species do not form spores, and Chase et al demonstrated clearer evidence for population growth and turnover than in our study, where extended dormancy seemed likely. This indicates that a greater number of generations experienced by an evolving population in situ may give rise to more recognizable evolutionary dynamics, but more work is needed to compare lab vs. soil-based evolution across comparable generational timeframes.

### Zinc limitation as a possible selective force for *P. megaterium* pre-deployment

The lack of strong post-burial evolutionary changes in our focal populations hindered our ability to assess ecologically-relevant selective forces at each site, as was our goal. However, the month-long lab incubation of *P. megaterium* in the carrier soil provided an example of the patterns we failed to see in the field. Of the mutations that rose to high frequencies during the lab incubation, the majority fell within genes related to zinc. While it is possible that this outcome occurred via purely stochastic processes, it suggests that zinc limitation could have been a strong selective pressure in the carrier soil pre-deployment. We were unable to find other studies documenting real-time evolutionary changes under extended periods of zinc limitation, and as such we could not assess whether our mutated genes—including components of a zinc transport system and a replacement for a zinc-dependent enzyme—are common evolutionary targets under zinc limitation. It was also beyond the scope of this study to assess whether these mutations improved zinc acquisition or *P. megaterium* fitness, but this is an intriguing avenue for further study.

Following on these results, we conducted a separate study replicating the lab-based carrier soil incubation in three different soils [56]. One of those soils came from the same location as the carrier soil here, with a similarly low level of zinc (1.6 ppm in Kaminsky, Burghardt [56] vs. 1.5 ppm in this study). Surprisingly, we did not observe zinc-related mutations in those populations. However, the soil in Kaminsky, Burghardt [56] was autoclaved for one additional round before inoculation with *P. megaterium* to further discourage the presence of non-focal species, and there was less evidence of contamination overall. We speculate that the presence of non-focal species in the carrier soil here may have made zinc even less bio-available than indicated by the soil analysis, and perhaps this competition drove our mutational patterns. This would agree with prior findings from media-based experimental evolution studies that evolution can proceed more rapidly in the face of competition from other taxa [18]. It would be a useful next step to compare evolutionary outcomes in two versions of the same soil – one cleared of most biota and one with residents undisturbed.

### Evaluation of the aChip as a tool for in situ experimental evolution and suggestions for improvement

Using the aChips, we were able to contain and re-capture populations of two different bacterial taxa in farm soil environments. We retrieved isolates two years after burial, in contrast to the sharp population decline often observed with direct inoculation of field soils [8, 57]. This indicates that aChip-like devices are promising tools for in situ study of soil microbes. However, we suggest several adjustments to aChip design to support greater population growth, tailor the selective pressures to which focal populations are exposed, and improve the potential for observable evolutionary changes.

First, using a larger plastic device that holds more carrier soil, such as the 24-well no-bottom plates employed in King, Kaminsky [58] (Greiner Bio-One, catalog: 662000–06), could support more population growth. However, the greater thickness of these devices could yield uneven exposure of cells to the exterior environment depending on their location within the wells, with soil adjacent to the membrane having the most interaction with resident microbes and interior soil having limited contact. Sampling of the aChips could account for this, potentially splitting carrier soil into membrane-adjacent vs. interior sub-samples to compare evolutionary outcomes. Alternatively, a simpler membrane bag like that used in Chase, Weihe [55] can also hold a larger volume of soil and may be simpler to construct and sample. However, the separate aChip wells could facilitate study of evolutionary dynamics at very fine spatial scales, if each well was treated as its own population and kept separate at the time of sampling.

Second, we suggest that the carrier soil for any given aChip should be sourced from the eventual burial site. This would allow microbial exposure to the abiotic properties of the burial soil itself rather than an arbitrary unrelated soil. This would also yield more easily interpretable results, as all possible selective pressures in and around the aChip would be sourced from the soil environment of interest. Depending on the evolving microbe of interest, filler options could also be sourced from other relevant environmental materials, such as plant litter [55]. However, we note that this would introduce a confounding variable – is there any influence from the environment outside of the aChip, or are the microbes merely responding to the conditions of the carrier soil *within* the aChip? Indeed, Chase, Weihe [55] also noted some mutations shared across populations evolving at different sites (similar to our shared mutations), which they attributed to adaptation to their carrier material. Additional controls would be required to tease this apart, for instance by burying a few replicate aChips completely sealed to the outside environment alongside those with a permeable membrane.

Third, the pre-incubation step in the carrier soil before aChip construction could be eliminated. Once the carrier soil is autoclaved, it could be loaded into half-constructed aChips and the focal microbes inoculated directly into the filled wells before the second membrane is affixed. This would guarantee a period of population growth within the aChips as the inoculated microbes expand into the cleared soil habitat, and more closely resembles the approach of the original ichip which aimed to contain a single cell within each well before burial [25].

Fourth, population bottlenecks could be imposed after aChip sampling points, by inoculating collected 100-isolate pools into fresh aChips with newly autoclaved carrier soil. Then, if carrying capacity had been reached inside the aChips, this could allow subsequent periods of population growth. A similar approach was used by Chase, Weihe [55]. Focusing on a non-spore forming microbe could also help.

Finally, we observed the presence of non-focal species in the aChip carrier soil both pre- and post-burial, largely stemming from the carrier soil itself. While this ultimately did not interfere with our ability to assess evolutionary change via the 100-isolate pools, additional rounds of sterilization of aChip carrier soil could alleviate this issue and enable mutation analysis directly from soil DNA extracts.

## Conclusions

With use of the aChips, we were able to secure and recapture evolving populations of *Priestia megaterium* and *S. lydicus* in soil environments out to two years at least. While evolutionary change post-burial was remarkably slow, the populations were not static and did accumulate some changes over the course of the experiment. Due to slower microbial growth in soil compared to lab media, it is likely that in situ adaptive evolution experiments need to run significantly longer to achieve a comparable number of generations as laboratory evolution experiments. With modifications to aChip design to enable more consistent population growth, it may be possible to decrease this evolutionary timeline. Such in situ experimental evolution experiments have the potential to yield valuable insights regarding microbial evolution in more real-world contexts.

## Supplementary Material

Supplementary_materials_ycag053

## Data Availability

The datasets generated during this current study are available in the NCBI SRA repository: https://www.ncbi.nlm.nih.gov/bioproject/?term=PRJNA933577, https://www.ncbi.nlm.nih.gov/bioproject/?term=PRJNA932007, https://www.ncbi.nlm.nih.gov/bioproject/?term=PRJNA936108.
