# Supplementary material for: The adaptation chip: repurposing the principles of the ichip for guiding in situ experimental evolution
Source: ISME Commun. 2026 Apr 3;6(1):ycag053. doi: 10.1093/ismeco/ycag053 (PMC13064666; doi:10.1093/ismeco/ycag053)
Supplement: Supplementary_materials_ycag053 [file supplementary_materials_ycag053.zip › FigureS5_Heatmap_Slyd_nonfocal.pdf]

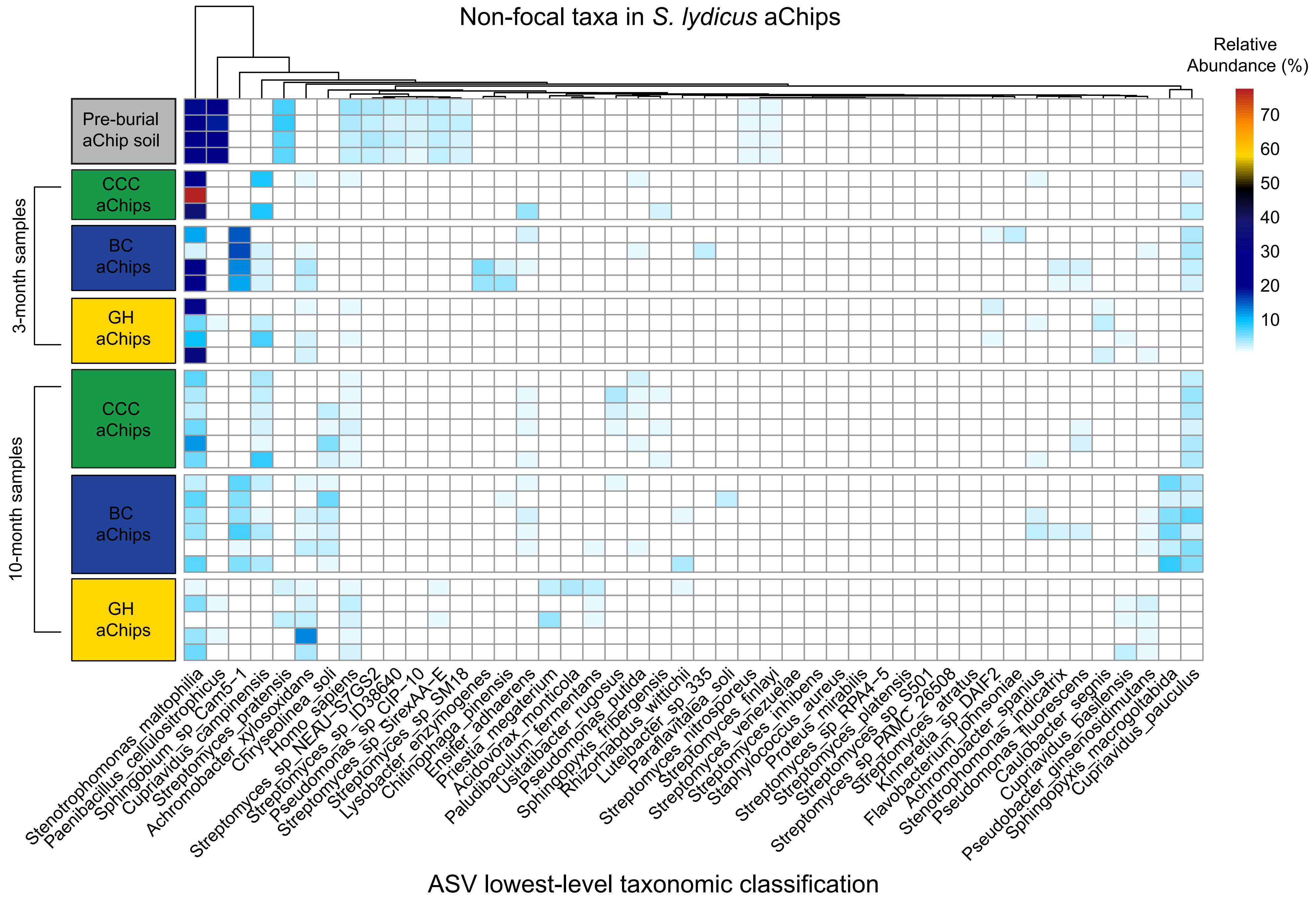

Non-focal taxa in S. lydicus aChips
Relative Abundance (%)
70
60
50
40
30
20
10
Pre-burial aChip soil
CCC aChips
BC aChips
GH aChips
3-month samples
10-month samples
Stenotrophomonas_maltophilia
Paenibacillus_cellulositrophicus
Sphingobium_sp_Cam5−1
Cupriavidus_campinensis
Streptomyces_pratensis
Achromobacter_xylosoxidans
Chryseolinea_soli
Homo_sapiens
Streptomyces_sp_NEAU−S7GS2
Streptomyces_sp_ID38640
Pseudomonas_sp_CIP−10
Streptomyces_sp_SirexAA−E
Streptomyces_sp_SM18
Lysobacter_enzymogenes
Chitinophaga_pinensis
Ensifer_adhaerens
Priestia_megaterium
Acidovorax_monticola
Paludibaculum_fermentans
Usitatibacter_rugosus
Pseudomonas_putida
Sphingopyxis_fribergensis
Rhizorhabdus_wittichii
Luteibacter_sp_335
Paraflavitalea_soli
Streptomyces_nitrosporeus
Streptomyces_finlayi
Streptomyces_venezuelae
Streptomyces_inhibens
Staphylococcus_aureus
Proteus_mirabilis
Streptomyces_sp_RPA4−5
Streptomyces_platensis
Streptomyces_sp_S501
Streptomyces_sp_PAMC_26508
Streptomyces_atratus
Kinneretia_sp_DAIF2
Flavobacterium_johnsoniae
Achromobacter_spanius
Stenotrophomonas_indicatrix
Pseudomonas_fluorescens
Caulobacter_segnis
Cupriavidus_basilensis
Pseudobacter_ginsenosidimutans
Sphingopyxis_macrogoltabida
Cupriavidus_pauculus
ASV lowest-level taxonomic classification
