# Supplementary material for: The adaptation chip: repurposing the principles of the ichip for guiding in situ experimental evolution
Source: ISME Commun. 2026 Apr 3;6(1):ycag053. doi: 10.1093/ismeco/ycag053 (PMC13064666; doi:10.1093/ismeco/ycag053)
Supplement: Supplementary_materials_ycag053 [file supplementary_materials_ycag053.zip › FigureS7_folE2_Zn_scatterplots.pdf]

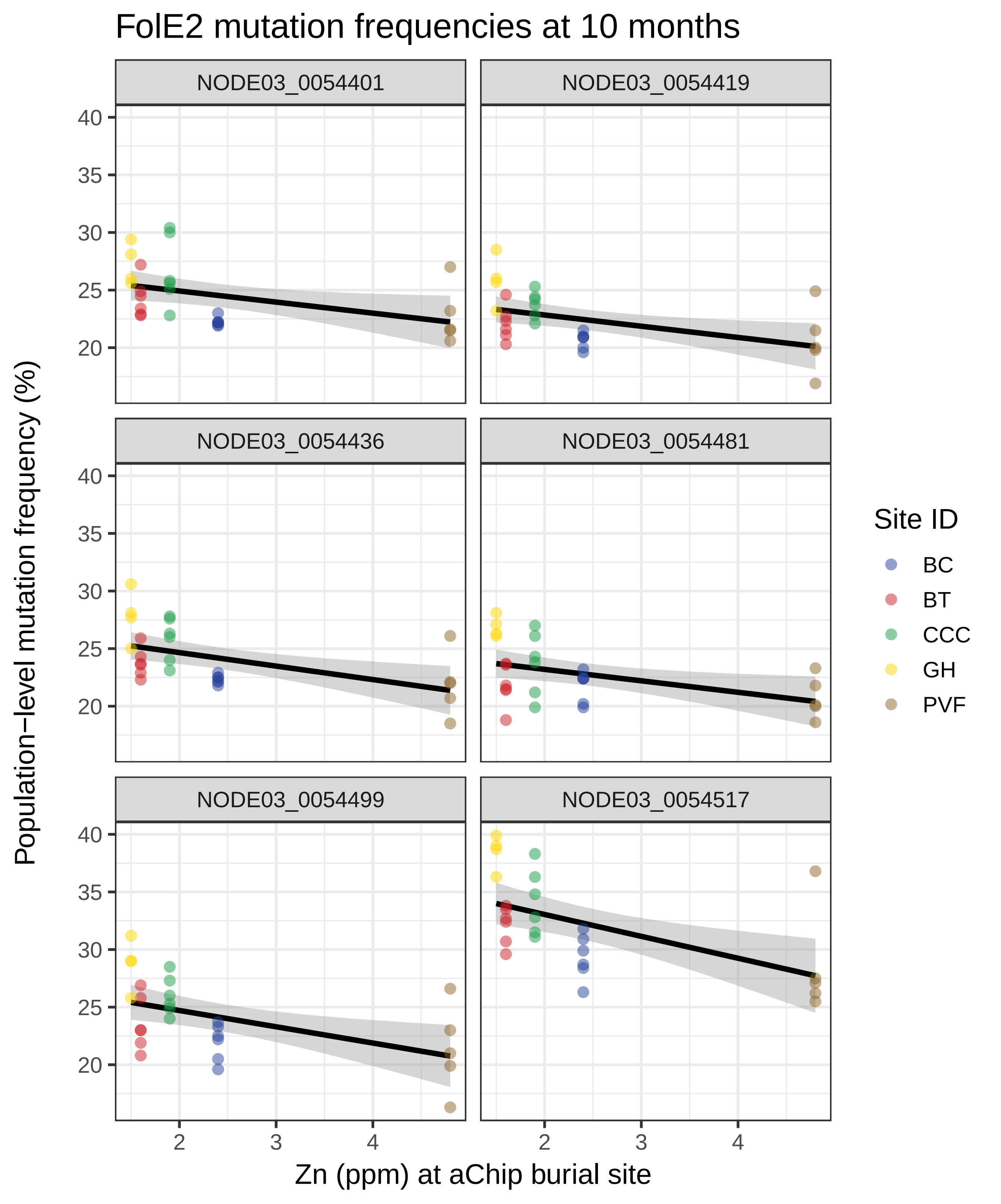

FolE2 mutation frequencies at 10 months
NODE03_0054401
NODE03_0054419
NODE03_0054436
NODE03_0054481
NODE03_0054499
NODE03_0054517
40
35
30
25
20
2
3
4
Population−level mutation frequency (%)
Zn (ppm) at aChip burial site
Site ID
BC
BT
CCC
GH
PVF
