# Supplementary material for: The adaptation chip: repurposing the principles of the ichip for guiding in situ experimental evolution
Source: ISME Commun. 2026 Apr 3;6(1):ycag053. doi: 10.1093/ismeco/ycag053 (PMC13064666; doi:10.1093/ismeco/ycag053)
Supplement: Supplementary_materials_ycag053 [file supplementary_materials_ycag053.zip › Table S1 - Soil properties.docx]

**Table S1**

| **Soil Property** | **Site ID** | | | | | |
| --- | --- | --- | --- | --- | --- | --- |
|  | **Carrier soil** | **BC** | **BT** | **CCC** | **GH** | **PVF** |
| Coordinates | 40.759969,  -77.880323 | 40.359130,  -77.288550 | 40.704054,  -76.739873 | 40.722370,  -77.928572 | 42.103339,  -79.552145 | 40.154991,  -76.352383 |
| Land Use | Monoculture corn | Vegetable bed | Fallow grassy area | Fallow grassy area | Pasture | Fallow grassy area |
| Soil type | Hagerstown silt loam | Melvin silt loam | Hartleton channery silt loam | Hagerstown silt loam | Chautauqua silt loam | Duffield silt loam |
| pH | 7.01 | 6.41 | 7.12 | 5.9 | 5.76 | 5.62 |
| Organic matter (%) | 4.95 | 4.87 | 2.49 | 2.2 | 4.59 | 3.56 |
| Nitrate (ppm) | 7.9 | 45.8 | 5.59 | 3.65 | 6.13 | 9.92 |
| Ammonium (ppm) | 224 | 2.41 | 0.84 | 1.27 | 1.39 | 1.42 |
| Phosphorus (ppm) | 52 | 15 | 15 | 28 | 21 | 16 |
| Potassium (ppm) | 182 | 375 | 40 | 106 | 37 | 59 |
| Magnesium (ppm) | 90.5 | 154 | 87 | 82 | 72 | 92 |
| Calcium (ppm) | 1156.6 | 1893.2 | 1625.6 | 956 | 910.6 | 986.5 |
| Zinc (ppm) | 1.5 | 2.4 | 1.6 | 1.9 | 1.5 | 4.8 |
| Copper (ppm) | 1.3 | 2.2 | 2.6 | 4 | 2.3 | 4.8 |
| Sulfur (ppm) | 25.2 | 11.7 | 7.1 | 10 | 16.8 | 14.8 |
