# Supplementary material for: The adaptation chip: repurposing the principles of the ichip for guiding in situ experimental evolution
Source: ISME Commun. 2026 Apr 3;6(1):ycag053. doi: 10.1093/ismeco/ycag053 (PMC13064666; doi:10.1093/ismeco/ycag053)
Supplement: Supplementary_materials_ycag053 [file supplementary_materials_ycag053.zip › Table S2 - Sample inventory.docx]

**Table S2**

| **Species** | **Time point** | **Sample type** | **Farm ID** | | | | | |
| --- | --- | --- | --- | --- | --- | --- | --- | --- |
|  |  |  | **Pre-burial** | **BC** | **BT** | **CCC** | **GH** | **PVF** |
| *P. megaterium* | 0mo | aChip soil | 4 | - | - | - | - | - |
|  | 3mo | aChip soil | - | 4 | 4 | 4 | 3 | 4 |
|  |  | 100-isolate pool | - | 4 | 4 | 3 | 1 | 4 |
|  | 10mo | aChip soil | - | 6 | 6 | 6 | 4 | 5 |
|  |  | 100-isolate pool | - | 5 | 6 | 6 | 4 | 5 |
|  | 24mo | aChip soil | - | 0 | 0 | 0 | 0 | 0 |
|  |  | 100-isolate pool | - | 5 | 0 | 6 | 5 | 0 |
|  | | | | | | | | |
| *S. lydicus* | 0mo | aChip soil | 4 | - | - | - | - | - |
|  | 3mo | aChip soil | - | 4 | 0 | 4 | 4 | 0 |
|  |  | 100-isolate pool | - | 3 | 0 | 2 | 4 | 0 |
|  | 10mo | aChip soil | - | 6 | 0 | 6 | 5 | 0 |
|  |  | 100-isolate pool | - | 5 | 0 | 6 | 3 | 0 |
|  | 24mo | aChip soil | - | 0 | 0 | 0 | 0 | 0 |
|  |  | 100-isolate pool | - | 6 | 0 | 5 | 3 | 0 |
