# Supplementary material for: The adaptation chip: repurposing the principles of the ichip for guiding in situ experimental evolution
Source: ISME Commun. 2026 Apr 3;6(1):ycag053. doi: 10.1093/ismeco/ycag053 (PMC13064666; doi:10.1093/ismeco/ycag053)
Supplement: Supplementary_materials_ycag053 [file supplementary_materials_ycag053.zip › Table S3 - ancestral genome quality.docx]

**Table S3**

| **Quality metric** | **Ancestral *P. megaterium* genome** | **Ancestral *S. lydicus* genome** |
| --- | --- | --- |
| **# contigs** | 73 | 110 |
| **Largest contig** | 1205537 | 752725 |
| **Total length** | 5488963 | 9083364 |
| **N50** | 823209 | 254658 |
| **Average coverage (%)** | 70.4% | 53.5% |
