# Supplementary material for: The adaptation chip: repurposing the principles of the ichip for guiding in situ experimental evolution
Source: ISME Commun. 2026 Apr 3;6(1):ycag053. doi: 10.1093/ismeco/ycag053 (PMC13064666; doi:10.1093/ismeco/ycag053)
Supplement: Supplementary_materials_ycag053 [file supplementary_materials_ycag053.zip › Table S7 - alignment scores formatted.docx]

| **Mutated gene copy**  **(and GC content)** | **Ancestral gene copy** | **GC content** | **Score (bits)** | **Expect value** | **Identities** | **Gaps** |
| --- | --- | --- | --- | --- | --- | --- |
| folE2 mutated vs.  (30.09%) | folE_1 | 38.14% | No significant alignment | | | |
|  | folE_2 | 41.09% | No significant alignment | | | |
|  | folE2 | 34.81% | No significant alignment | | | |
|  | | | | | | |
| ftsW_3 mutated vs.  (37.36%) | ftsW_1 | 38.12% | No significant alignment | | | |
|  | ftsW_2 | 40.87% | No significant alignment | | | |
|  | ftsW_3 | 35.85% | 1055 | 0 | 589/ 598 (98%) | 0/598 (0%) |
|  | | | | | | |
| psiE_2 mutated vs.  (34.72%) | psiE_1 | 26.71% | No significant alignment | | | |
|  | psiE_2 | 32.14% | 259 | 3.00E-72 | 142/143 (99%) | 0/143 (0%) |
|  | | | | | | |
| yndE_5 mutated vs.  (36.36%) | yndE_1 | 34.42% | No significant alignment | | | |
|  | yndE_2 | 36.32% | No significant alignment | | | |
|  | yndE_3 | 38.10% | No significant alignment | | | |
|  | yndE_4 | 38.11% | No significant alignment | | | |
|  | yndE_5 | 37.06% | 405 | 6.00E-116 | 233/240 (97%) | 0/240(0%) |
|  | | | | | | |
| znuA_2 mutated vs.  (34.54%) | znuA_1 | 36.85% | No significant alignment | | | |
|  | znuA_2 | 37.58% | 658 | 0 | 414/443 (93%) | 0/443 (0%) |
|  | znuA_3 | 33.02% | 228 | 3.00E-62 | 271/339 (80%) | 23/339 (7%) |
|  | | | | | | |
| znuA_3 mutated vs.  (30.27%) | znuA_1 | 36.85% | No significant alignment | | | |
|  | znuA_2 | 37.58% | No significant alignment | | | |
|  | znuA_3 | 33.02% | 270 | 3.00E-75 | 184/203 (91%) | 0/203 (0%) |
|  | | | | | | |
| znuC_2 mutated vs.  (36.71%) | znuC_1 | 37.31% | No significant alignment | | | |
|  | znuC_2 | 35.19% | No significant alignment | | | |
|  | znuC_3 | 36.86% | 324 | 2.00E-91 | 269/316 (85%) | 0/316 (0%) |
|  | | | | | | |
| znuC_3 mutated vs.  (35.48%) | znuC_1 | 37.31% | No significant alignment | | | |
|  | znuC_2 | 35.19% | No significant alignment | | | |
|  | znuC_3 | 36.86% | 686 | 0 | 457/500 (91%) | 0/500 (0%) |
