# Supplementary material for: The adaptation chip: repurposing the principles of the ichip for guiding in situ experimental evolution
Source: ISME Commun. 2026 Apr 3;6(1):ycag053. doi: 10.1093/ismeco/ycag053 (PMC13064666; doi:10.1093/ismeco/ycag053)
Supplement: Supplementary_materials_ycag053 [file supplementary_materials_ycag053.zip › Table S9 - Slyd shared mutation annotations formatted.docx]

| **Gene ID** | **Gene Annotation** |  | **Gene ID** | **Gene Annotation** |
| --- | --- | --- | --- | --- |
| gene_00211 | Hypothetical protein |  | gene_04037 | IS5 family transposase |
| gene_00231 | VOC family protein |  | gene_04039 | IS481 family transposase |
| gene_00247 | Hypothetical protein |  | gene_04276 | Hypothetical protein |
| gene_00258 | Hypothetical protein |  | gene_04479 | Polyprenyl synthetase family protein |
| gene_00402 | MFS transporter |  | gene_04729 | sugar ABC transporter ATP-binding protein |
| gene_00795 | ARAC family transcriptional regulator |  | gene_04966 | Hypothetical protein |
| gene_00935 | Glycosyl transferase |  | gene_05152 | Hypothetical protein |
| gene_00963 | Hypothetical protein |  | gene_05194 | Permease |
| gene_01298 | ATP-binding protein |  | gene_05665 | Transcriptional regulator |
| gene_01301 | Hypothetical protein |  | gene_05759 | Phosphatase |
| gene_01452 | Protease |  | gene_05827 | Nicotinate phosphoribosyltransferase |
| gene_01644 | LysR family transcriptional regulator |  | gene_05898 | CDP-glyverol:glycerophosphate glycerophosphotransferase |
| recD2 | ATP-dependent RecD-like DNA helicase |  | gene_05989 | 1-acyl-sn-glycerol-3-phosphate acyltransferase |
| gene_01907 | Aspartate carbamoyltransferase |  | gene_06028 | TetR family transcriptional regulator |
| gene_02171 | ABC transporter substrate-binding protein |  | gene_06039 | ABC transporter substrate-binding protein |
| gene_02403 | ATP-binding protein |  | gene_06116 | Phosphotriesterase |
| gene_02465 | Hypothetical protein |  | gene_06180 | IS1182 family transposase ISAcma46 |
| gene_02511 | TetR family transcriptional regulator |  | gene_06310 | Hypothetical protein |
| gene_02591 | Glycosyl transferase |  | gene_06488 | Phosphoenolpyruvate synthase |
| gene_02747 | Hypothetical protein |  | gene_06529 | 2-phospho-L-lactate transferase |
| gene_02800 | Transporter |  | dltA | D-alanine-D-alanyl carrier protein ligase |
| gene_02879 | Hypothetical protein |  | gene_06841 | non-ribosomal peptide synthetase/polyketide synthase |
| gene_02945 | Polyketide synthase |  | gene_06945 | Hypothetical protein |
| blsF | CGA synthase-related protein |  | gene_07037 | Hypothetical protein |
| gene_03059 | beta-ACP synthase |  | gene_07052 | Hypothetical protein |
| gene_03521 | Hypothetical protein |  | gene_07146 | Serine/threonine protein kinase |
| gene_03555 | cell division protein FtsK |  | gene_07163 | ABC transporter ATP-binding protein |
| gene_03563 | MFS transporter |  | gene_07200 | HAD family hydrolase |
| gene_03737 | Hypothetical protein |  | gene_07311 | Pyruvate, phosphate dikinase |
| hpt | Hypoxanthine phosphoribosyltransferase |  | gene_07326 | Bcr/CflA family drug resistance efflux transporter |
| gene_03814 | coenzyme F420 biosynthesis-associated protein |  | gene_07480 | IS5 family transposase |
| gene_03883 | non-ribosomal peptide synthetase |  | gene_07507 | Hypothetical protein |
| gene_03935 | Hypothetical protein |  | gene_07546 | Hypothetical protein |
| gene_03939 | Hypothetical protein |  | gene_07864 | Pyridine nucleotide-disulfide oxidoreductase |
