# Supplementary material for: The adaptation chip: repurposing the principles of the ichip for guiding in situ experimental evolution
Source: ISME Commun. 2026 Apr 3;6(1):ycag053. doi: 10.1093/ismeco/ycag053 (PMC13064666; doi:10.1093/ismeco/ycag053)
Supplement: Supplementary_materials_ycag053 [file supplementary_materials_ycag053.zip › aChip_ISMEComms_Supplemental_FINAL_CLEAN.docx]

**SUPPLEMENTAL FIGURES AND TABLES**

**Figure S1:** Methods and bioinformatics summary.


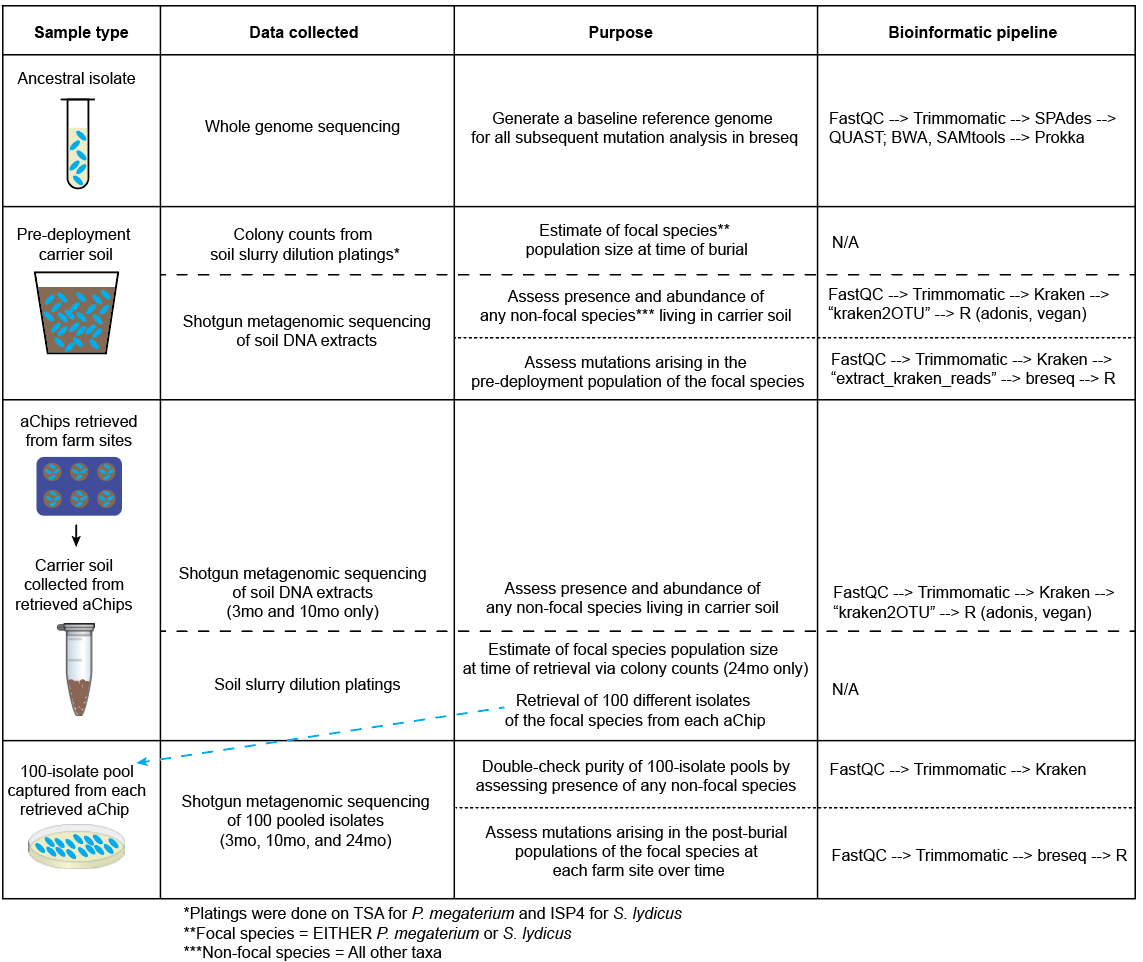


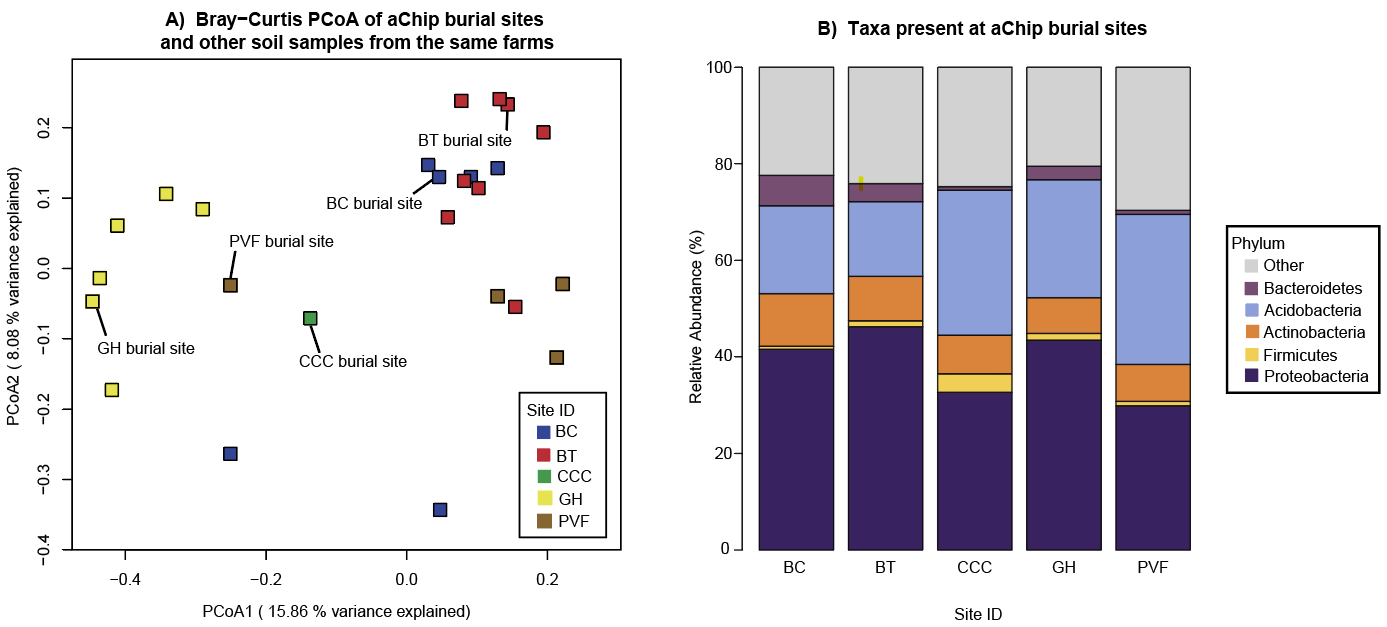


**Figure S2:** Resident microbes at burial sites. A) PCoA of Bray-Curtis distances between bacterial ASV communities at aChip burial sites and other soil samples from the same farms as determined by 16S rRNA gene sequencing. B) Relative abundance of dominant phyla at each aChip burial site as determined by 16S rRNA gene sequencing. DNA was extracted from ~250 mg of soil from each sample with the NucleoSpin Soil 96 kit (Macherey-Nagel, Düren, Germany). Initial 16S rRNA gene PCR reactions were carried out using the universal bacterial primers 341F (5’- CCTACGGGNGGCWGCAG-3′) and 806R (5′- GGACTACHVGGGTWTCTAAT-3′), and subsequent library preparation was conducted as described previously (Kaminsky et al 2021). Raw sequences were processed into ASV tables using DADA2 (Callahan et al 2016). PCoA analysis was conducted in R v4.2.1 using the package vegan.


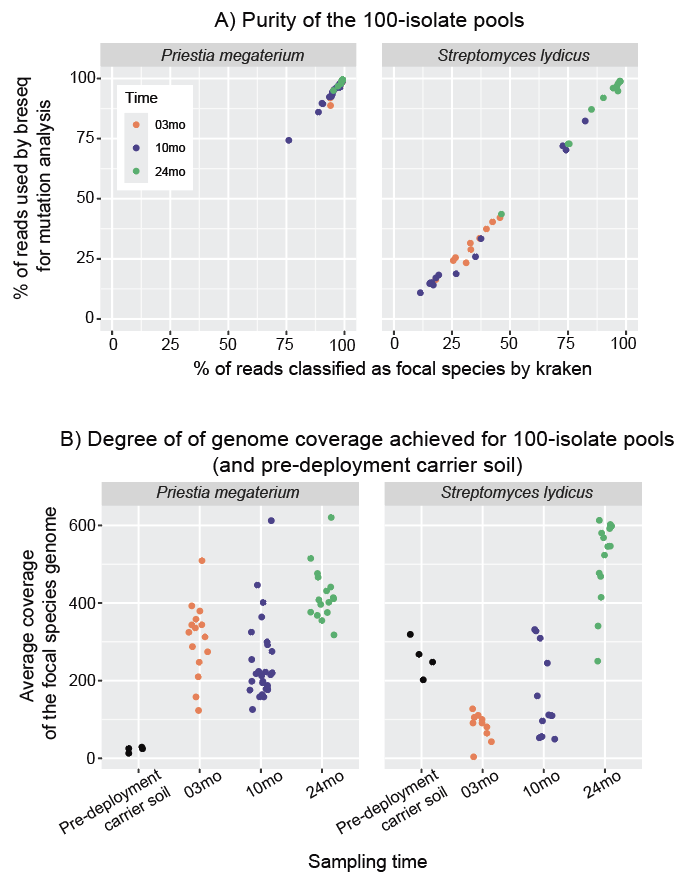


**Figure S3:** Quality metrics for the 100-isolate pools used for mutation analysis in breseq. Each point represents one 100-isolate pool, which are colored by sampling time. A) Purity of the 100-isolate *P. megaterium* and *S. lydicus* pools as reported by kraken and breseq, showing good agreement between the two softwares. B) Average depth of coverage of the ancestral genome achieved for each 100-isolate pool. Values were calculated from the number of reads assigned as the focal species by kraken compared to the length of the ancestral genome.

**Figure S4:** Heatmap of non-focal taxa inside *P. megaterium* aChips across the course of the experiment. This figure only includes the top 20 most abundant taxa from the pre-burial aChip soil, plus any additional taxa with at least 2% relative abundance in at least one aChip. Reads were rarefied to 2717799 reads per sample before calculating relative abundance.


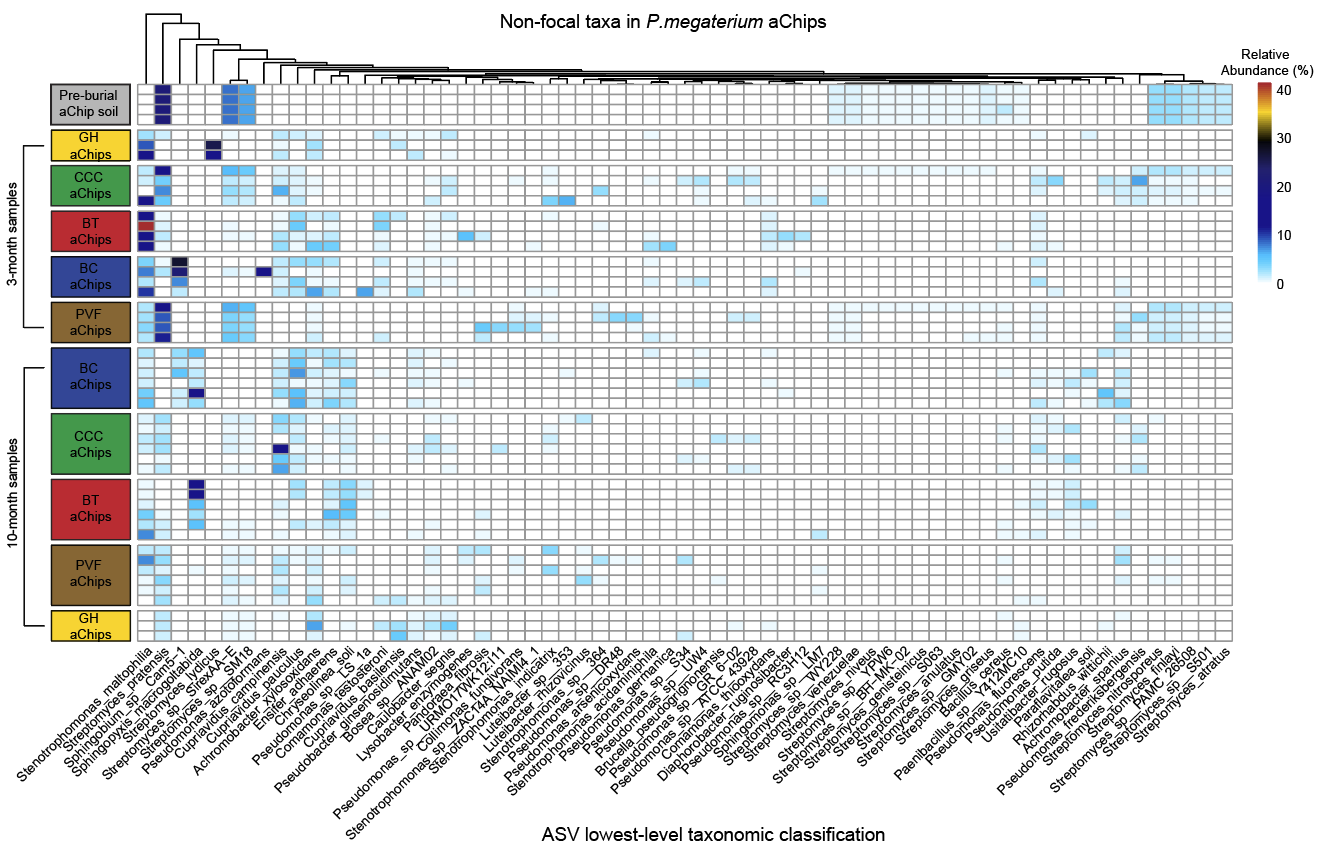


**Figure S5:** Heatmap of non-focal taxa inside *S. lydicus* aChips across the course of the experiment. This figure only includes the top 20 most abundant taxa from the pre-burial aChip soil, plus any additional taxa with at least 2% relative abundance in at least one aChip. Reads were rarefied to 1486894 reads per sample before calculating relative abundance.


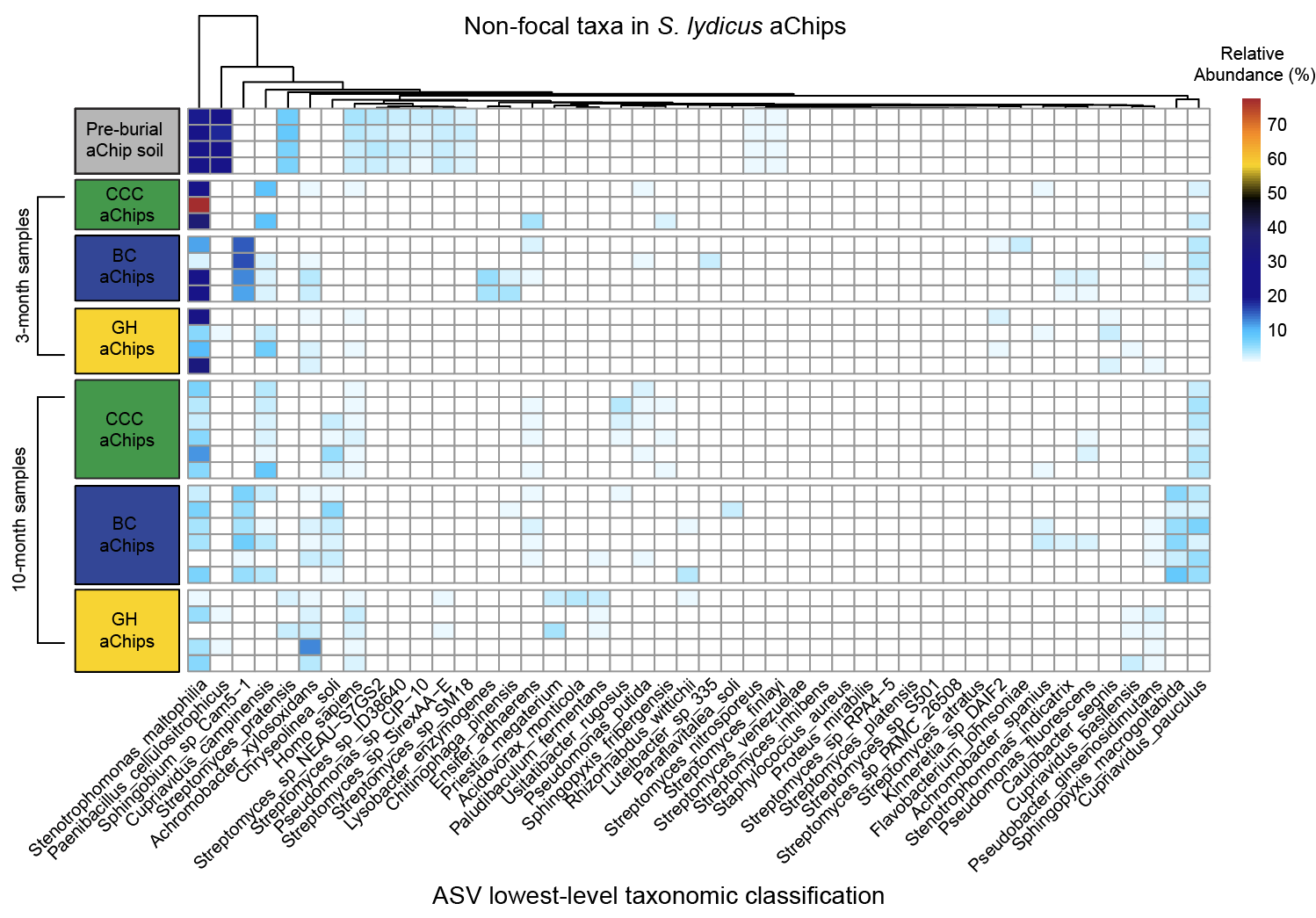


**Figure S6:** Example of mutation linkage in a gene carrying mutations pre-deployment. Displayed is a screenshot of breseq evidence for a single mutation (see dash) in gene znuA_2 from the ancestral carrier soil shotgun metagenomic sequencing data, demonstrating that this mutation is located on the same reads as other nearby mutations.


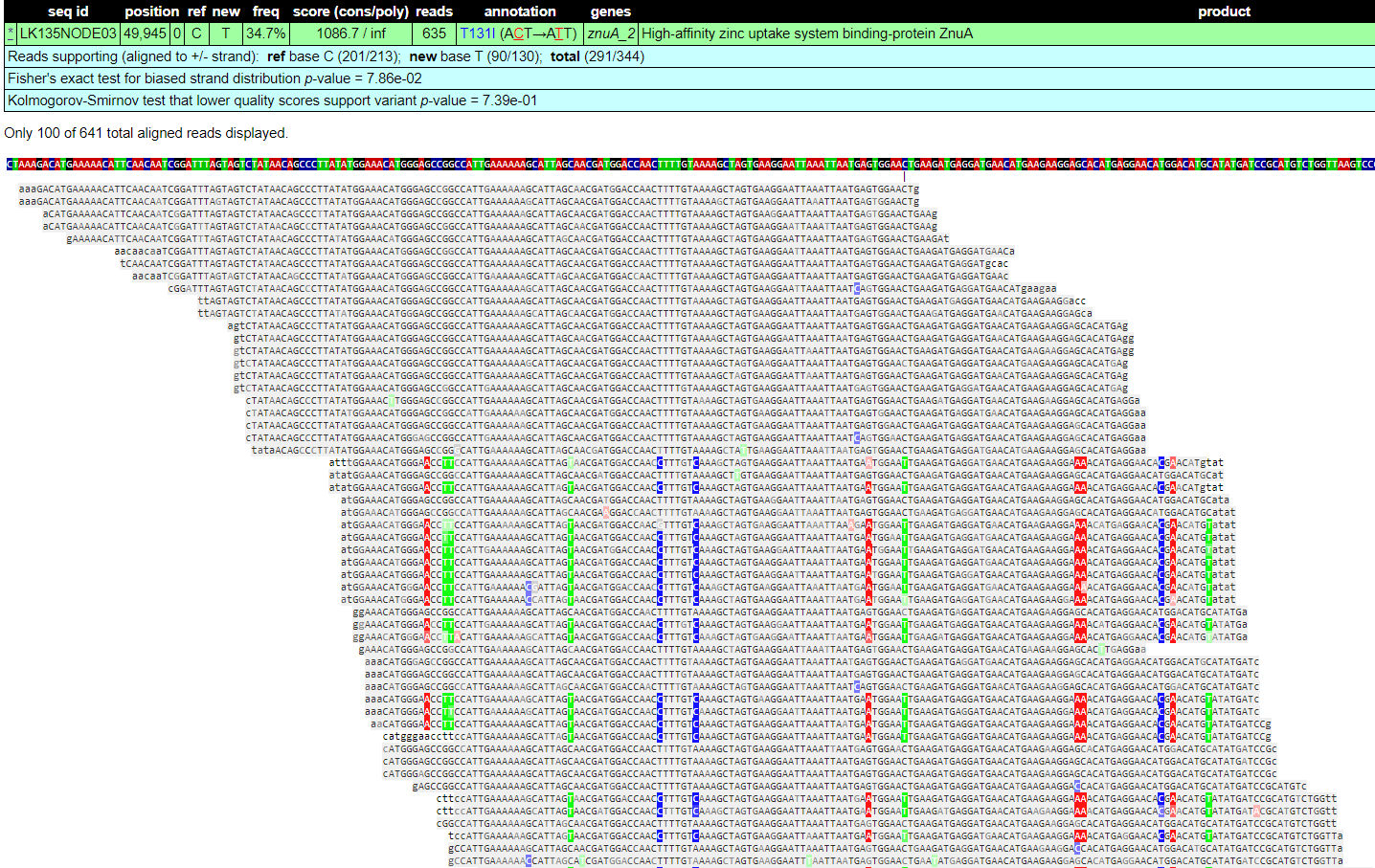


**
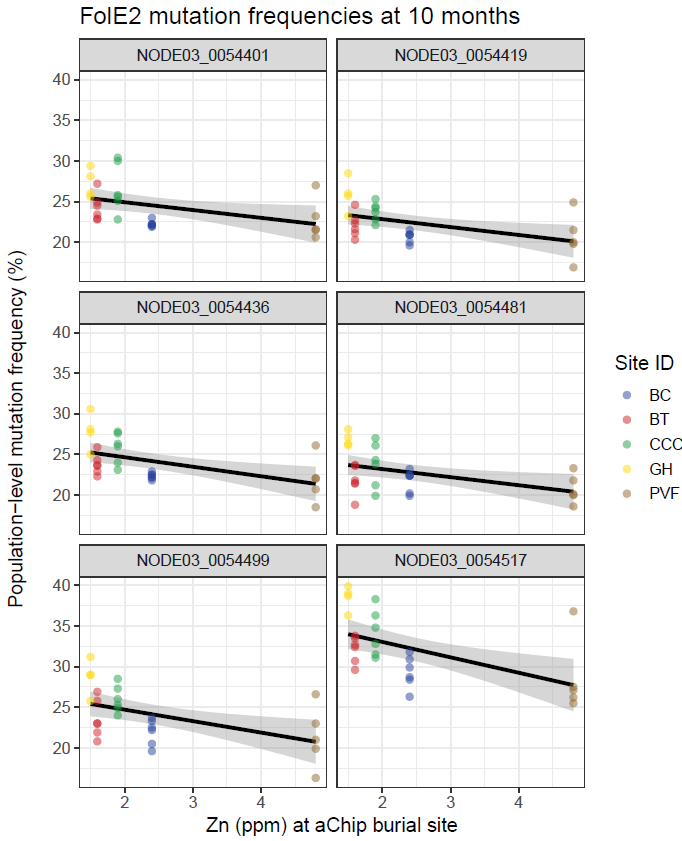
**

**Figure S7:** Scatterplots showing significant Pearson correlations between zinc levels in the aChip burial soil and frequencies of shared non-synonymous mutations in folE2 (see **Table S8**).

**
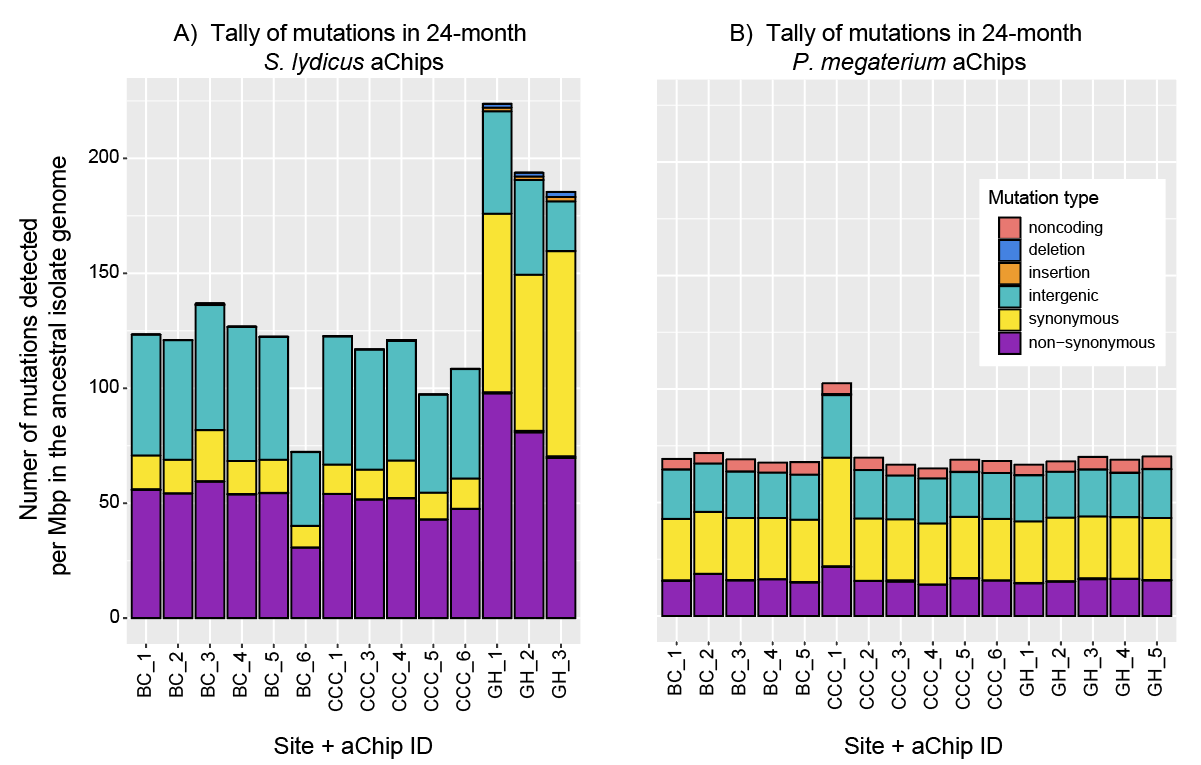
**

**Figure S8:** Number of mutations detected in each individual aChip collected at the 24-month time point for A) S. lydicus and B) P. megaterium. Mutation counts are normalized to the length of the appropriate ancestral genome.

**Table S1:** Abiotic properties of the carrier soil used for aChip construction and of the soils at the aChip burial sites, as measured by the Penn State Agricultural Analytical Services Lab. pH was determined in a 1:1 water to soil suspension. Organic matter was determined by mass loss on combustion. Water content was determined by mass loss after 24h of drying at 70ºC. Nitrate content was determined by specific ion electrodes. All other nutrient contents were determined with Mehlich 3 extractables.

| **Soil Property** | **Site ID** | | | | | |
| --- | --- | --- | --- | --- | --- | --- |
|  | **Carrier soil** | **BC** | **BT** | **CCC** | **GH** | **PVF** |
| Coordinates | 40.759969,  -77.880323 | 40.359130,  -77.288550 | 40.704054,  -76.739873 | 40.722370,  -77.928572 | 42.103339,  -79.552145 | 40.154991,  -76.352383 |
| Land Use | Monoculture corn | Vegetable bed | Fallow grassy area | Fallow grassy area | Pasture | Fallow grassy area |
| Soil type | Hagerstown silt loam | Melvin silt loam | Hartleton channery silt loam | Hagerstown silt loam | Chautauqua silt loam | Duffield silt loam |
| pH | 7.01 | 6.41 | 7.12 | 5.9 | 5.76 | 5.62 |
| Organic matter (%) | 4.95 | 4.87 | 2.49 | 2.2 | 4.59 | 3.56 |
| Nitrate (ppm) | 7.9 | 45.8 | 5.59 | 3.65 | 6.13 | 9.92 |
| Ammonium (ppm) | 224 | 2.41 | 0.84 | 1.27 | 1.39 | 1.42 |
| Phosphorus (ppm) | 52 | 15 | 15 | 28 | 21 | 16 |
| Potassium (ppm) | 182 | 375 | 40 | 106 | 37 | 59 |
| Magnesium (ppm) | 90.5 | 154 | 87 | 82 | 72 | 92 |
| Calcium (ppm) | 1156.6 | 1893.2 | 1625.6 | 956 | 910.6 | 986.5 |
| Zinc (ppm) | 1.5 | 2.4 | 1.6 | 1.9 | 1.5 | 4.8 |
| Copper (ppm) | 1.3 | 2.2 | 2.6 | 4 | 2.3 | 4.8 |
| Sulfur (ppm) | 25.2 | 11.7 | 7.1 | 10 | 16.8 | 14.8 |

**Table S2:** Number of samples sequenced for each farm site and time point. Initially both soil DNA and 100-isolate pool DNA was sequenced, but for the final time point only 100-isolate pools were sequenced. For all *S. lydicus* samples and for *P. megaterium* 24 month samples, only those from three of the five farm sites were sequenced due to cost.

| **Species** | **Time point** | **Sample type** | **Farm ID** | | | | | |
| --- | --- | --- | --- | --- | --- | --- | --- | --- |
|  |  |  | **Pre-burial** | **BC** | **BT** | **CCC** | **GH** | **PVF** |
| *P. megaterium* | 0mo | aChip soil | 4 | - | - | - | - | - |
|  | 3mo | aChip soil | - | 4 | 4 | 4 | 3 | 4 |
|  |  | 100-isolate pool | - | 4 | 4 | 3 | 1 | 4 |
|  | 10mo | aChip soil | - | 6 | 6 | 6 | 4 | 5 |
|  |  | 100-isolate pool | - | 5 | 6 | 6 | 4 | 5 |
|  | 24mo | aChip soil | - | 0 | 0 | 0 | 0 | 0 |
|  |  | 100-isolate pool | - | 5 | 0 | 6 | 5 | 0 |
|  | | | | | | | | |
| *S. lydicus* | 0mo | aChip soil | 4 | - | - | - | - | - |
|  | 3mo | aChip soil | - | 4 | 0 | 4 | 4 | 0 |
|  |  | 100-isolate pool | - | 3 | 0 | 2 | 4 | 0 |
|  | 10mo | aChip soil | - | 6 | 0 | 6 | 5 | 0 |
|  |  | 100-isolate pool | - | 5 | 0 | 6 | 3 | 0 |
|  | 24mo | aChip soil | - | 0 | 0 | 0 | 0 | 0 |
|  |  | 100-isolate pool | - | 6 | 0 | 5 | 3 | 0 |

**Table S3:** Quality of the *de novo* assembled ancestral isolate genomes used as the baseline for the mutation analysis.

| **Quality metric** | **Ancestral *P. megaterium* genome** | **Ancestral *S. lydicus* genome** |
| --- | --- | --- |
| **# contigs** | 73 | 110 |
| **Largest contig** | 1205537 | 752725 |
| **Total length** | 5488963 | 9083364 |
| **N50** | 823209 | 254658 |
| **Average coverage (%)** | 70.4% | 53.5% |

**
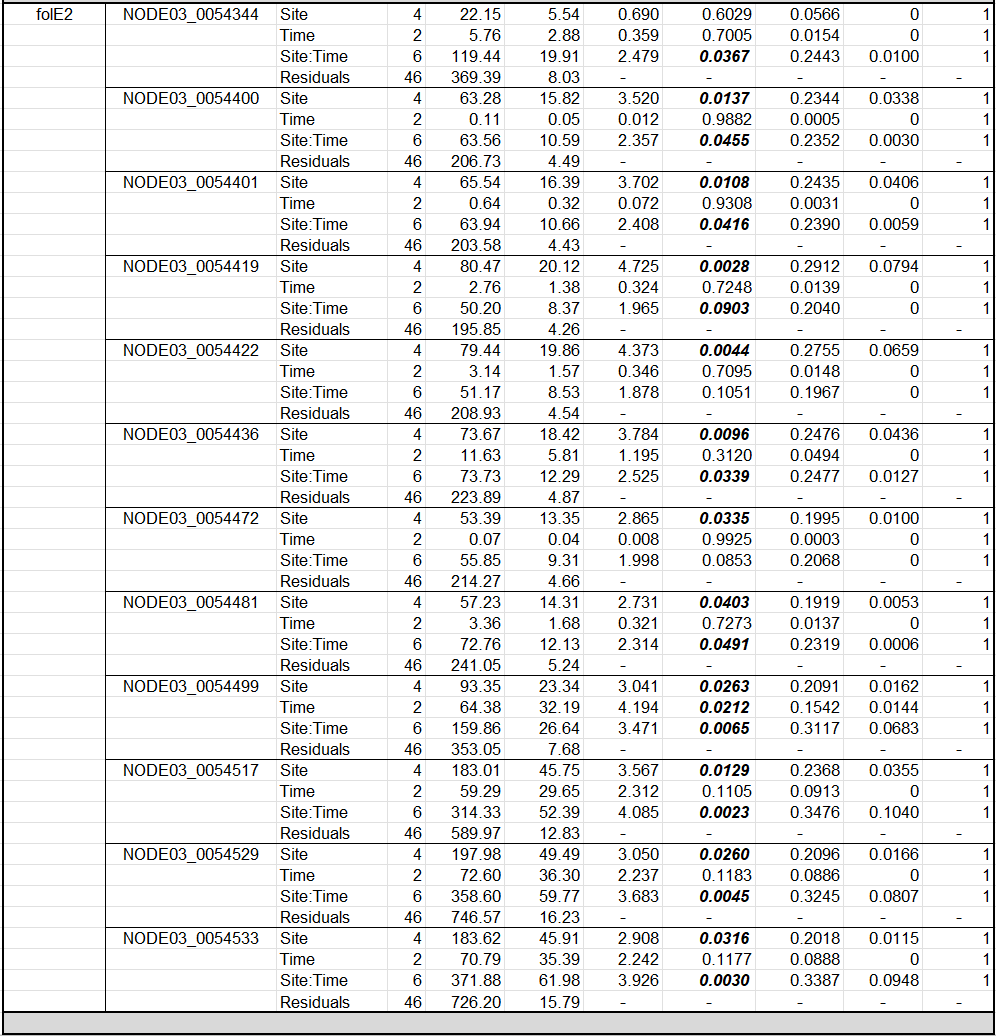

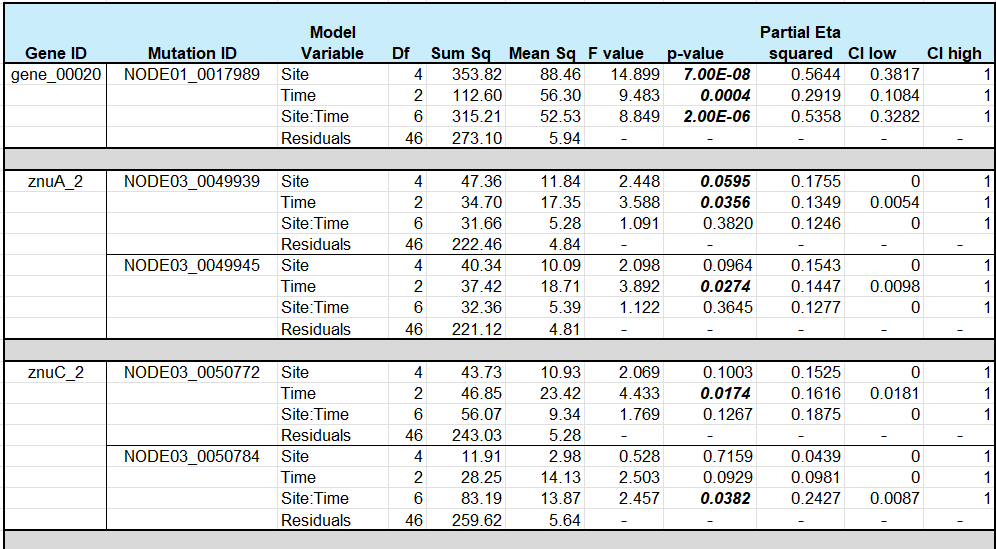
Table S4:** Two-way ANOVA model results testing the frequencies of the *P. megaterium* non-synonymous shared mutations on the basis of burial site, harvest time, and site*time interaction.

**
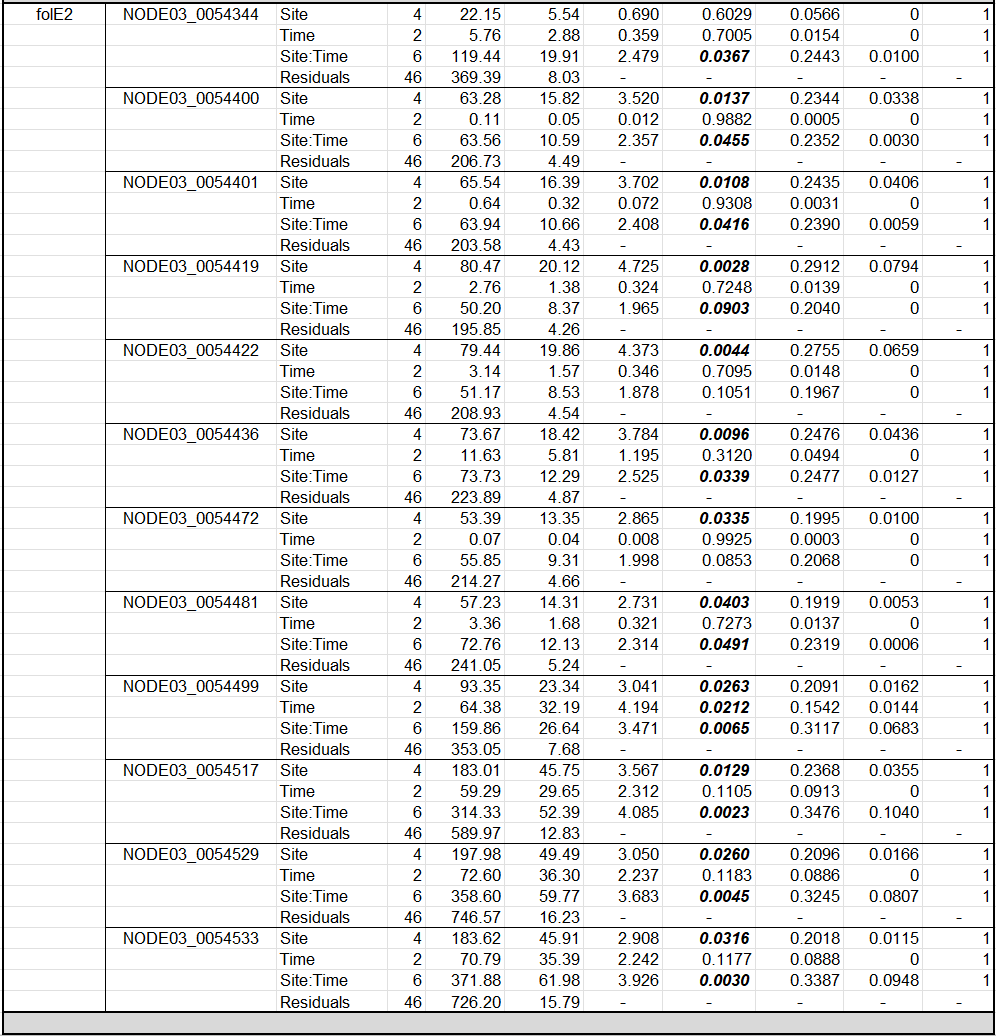

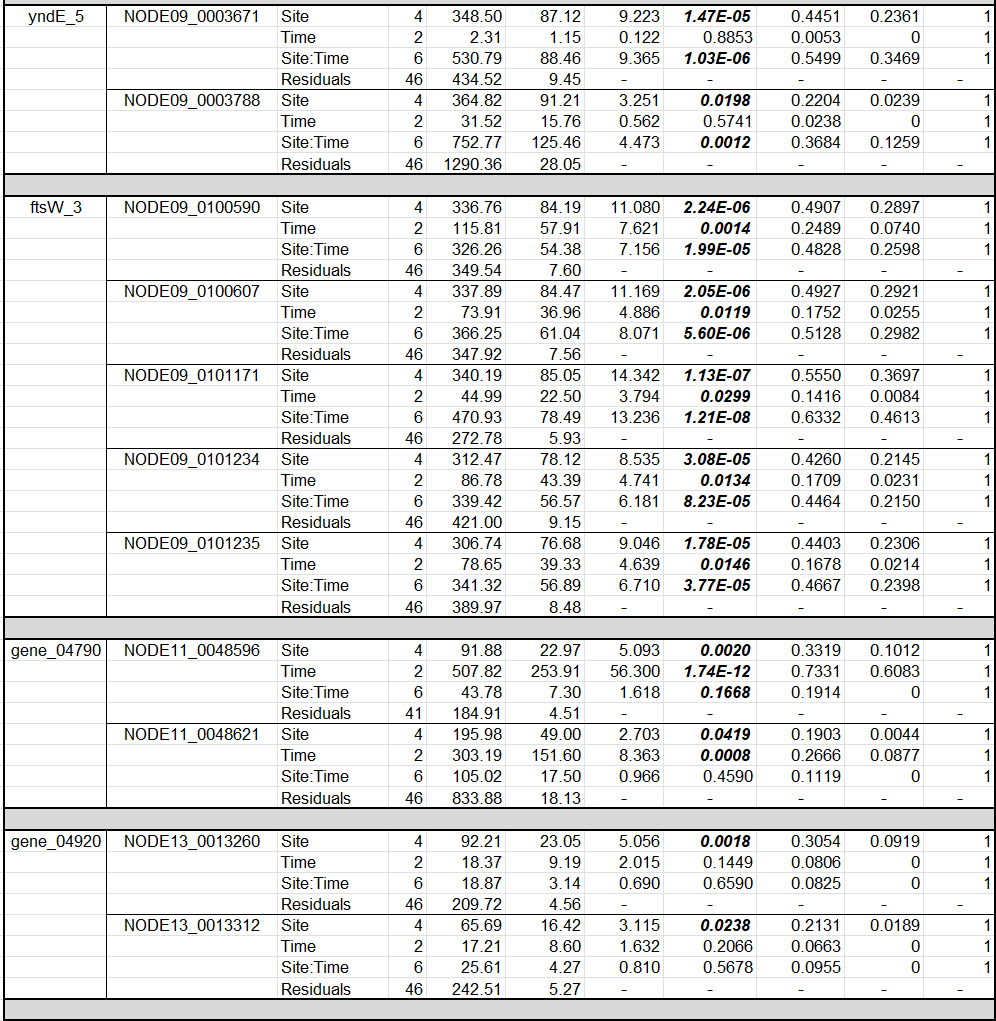
**

**
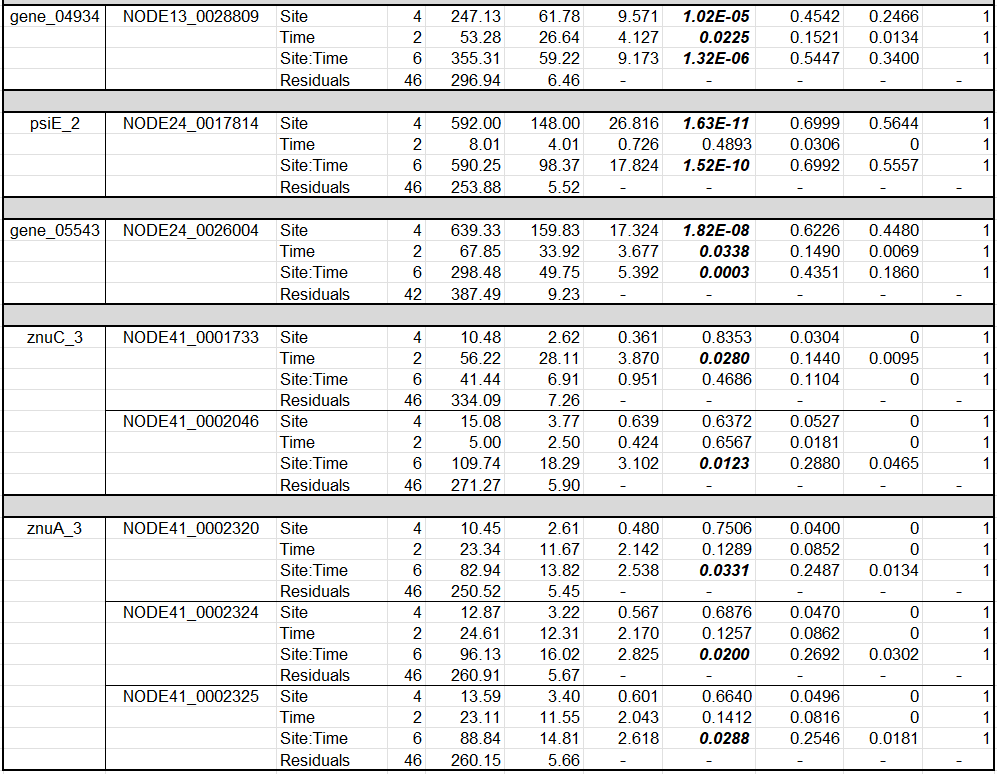
**

**Table S5:** Results of Tukey’s HSD post-hoc tests conducted on mutations with a significant Site*Time interaction in a two-way ANOVA model (see **Table S4**). Here we compare different sites at the same time point, with significant p-values marked in green, and only include mutations with at least one significant pairwise comparison. These mutations are also marked with an asterisk in Figure 4.

**
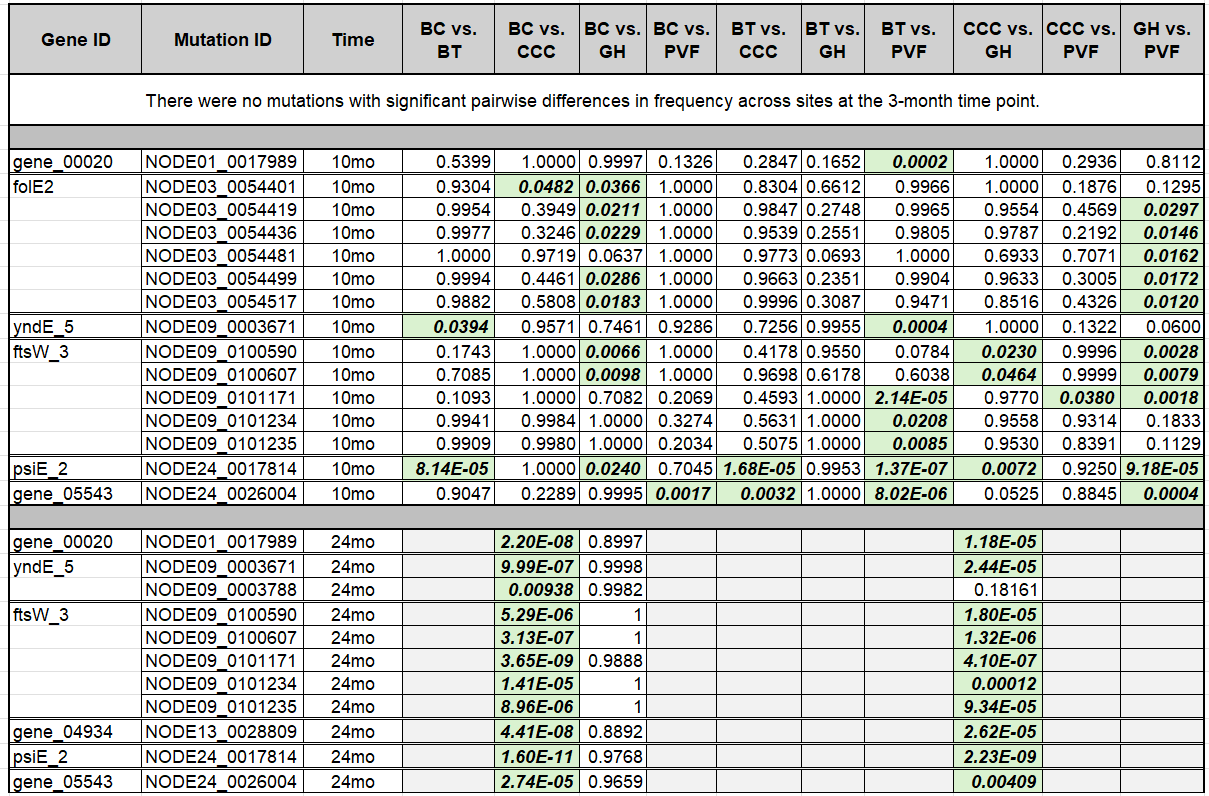
**

**Table S6:** Results of Tukey’s HSD post-hoc tests conducted on mutations with a significant Site*Time interaction in a two-way ANOVA model (see **Table S4**). Here we compare across time points within one site, with significant p-values marked in green, and only include mutations with at least one significant pairwise comparison.

**
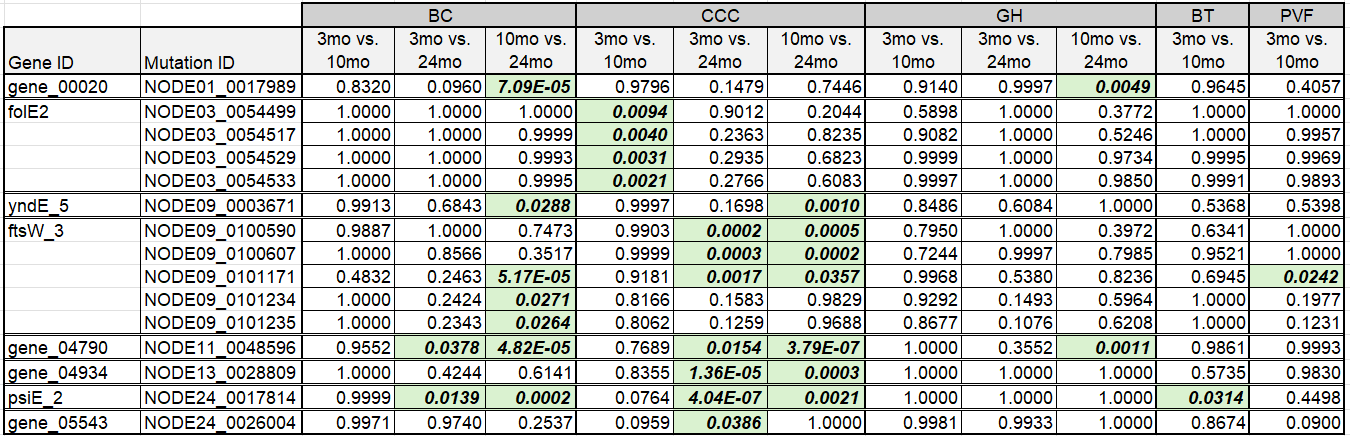
**

**Table S7:** Alignment scores for mutated versions of the genes carrying shared *P. megaterium* mutations vs. each copy of that gene in the ancestral genome. Also listed is the GC content for mutated vs. ancestral gene copies. Scores were calculated by creating a local database of ancestral gene copies then using BLAST to check alignments of mutated versions of each gene.


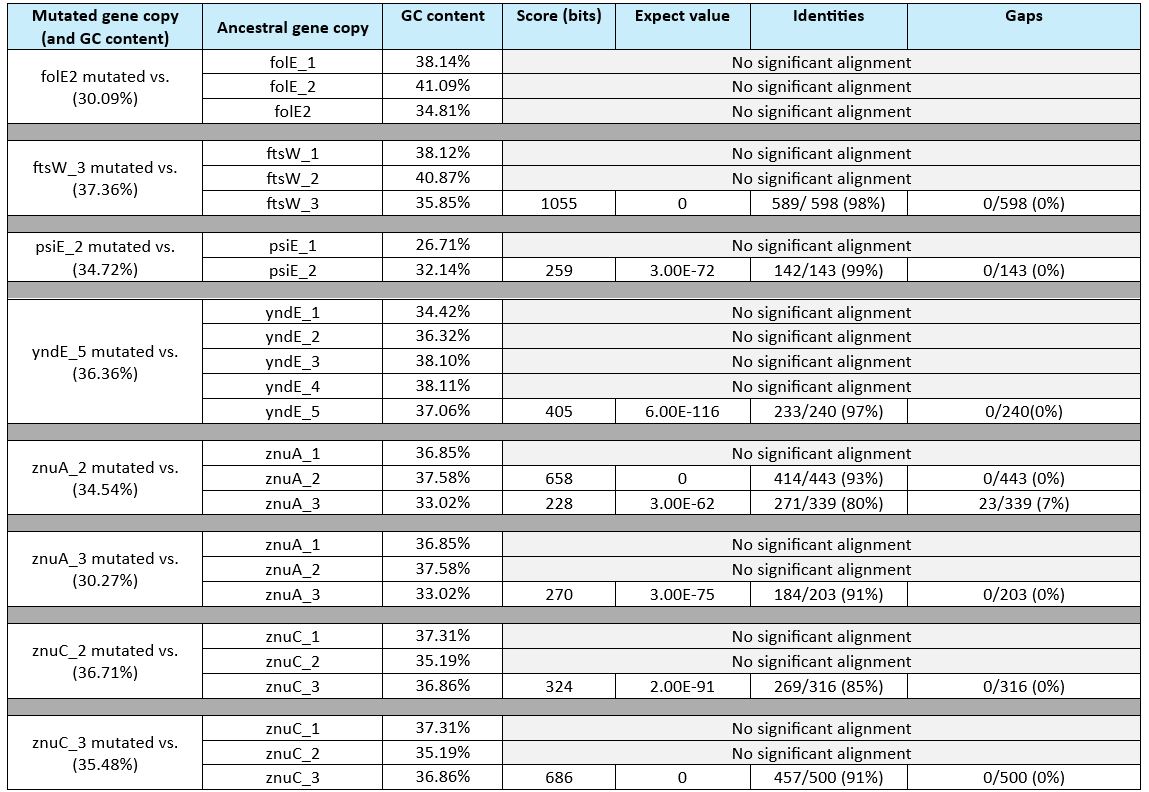


**Table S8:** Pearson correlations between select burial site abiotic features and shared mutation frequencies with a significant pairwise difference by site according to two-way ANOVA and Tukey’s HSD (see **Table S5**). Significant correlations have a bold italic p-value and the correlation coefficient is colored green for positive correlations and red for negative correlations.


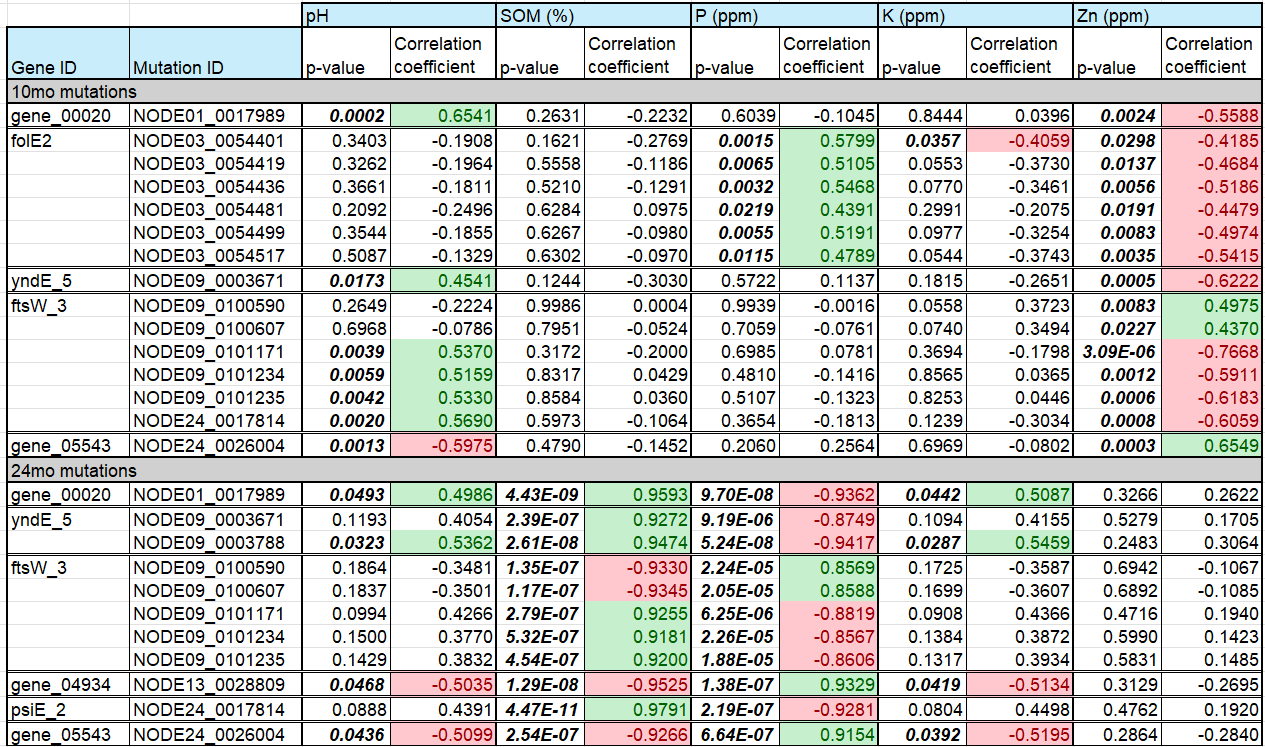


**Table S9**: Gene annotations for the 112 shared non-synonymous mutations in *S. lydicus* 100-isolate pools at the 24-month sampling point, displayed in **Figure 6**.

| **Gene ID** | **Gene Annotation** |  | **Gene ID** | **Gene Annotation** |
| --- | --- | --- | --- | --- |
| gene_00211 | Hypothetical protein |  | gene_04037 | IS5 family transposase |
| gene_00231 | VOC family protein |  | gene_04039 | IS481 family transposase |
| gene_00247 | Hypothetical protein |  | gene_04276 | Hypothetical protein |
| gene_00258 | Hypothetical protein |  | gene_04479 | Polyprenyl synthetase family protein |
| gene_00402 | MFS transporter |  | gene_04729 | sugar ABC transporter ATP-binding protein |
| gene_00795 | ARAC family transcriptional regulator |  | gene_04966 | Hypothetical protein |
| gene_00935 | Glycosyl transferase |  | gene_05152 | Hypothetical protein |
| gene_00963 | Hypothetical protein |  | gene_05194 | Permease |
| gene_01298 | ATP-binding protein |  | gene_05665 | Transcriptional regulator |
| gene_01301 | Hypothetical protein |  | gene_05759 | Phosphatase |
| gene_01452 | Protease |  | gene_05827 | Nicotinate phosphoribosyltransferase |
| gene_01644 | LysR family transcriptional regulator |  | gene_05898 | CDP-glyverol:glycerophosphate glycerophosphotransferase |
| recD2 | ATP-dependent RecD-like DNA helicase |  | gene_05989 | 1-acyl-sn-glycerol-3-phosphate acyltransferase |
| gene_01907 | Aspartate carbamoyltransferase |  | gene_06028 | TetR family transcriptional regulator |
| gene_02171 | ABC transporter substrate-binding protein |  | gene_06039 | ABC transporter substrate-binding protein |
| gene_02403 | ATP-binding protein |  | gene_06116 | Phosphotriesterase |
| gene_02465 | Hypothetical protein |  | gene_06180 | IS1182 family transposase ISAcma46 |
| gene_02511 | TetR family transcriptional regulator |  | gene_06310 | Hypothetical protein |
| gene_02591 | Glycosyl transferase |  | gene_06488 | Phosphoenolpyruvate synthase |
| gene_02747 | Hypothetical protein |  | gene_06529 | 2-phospho-L-lactate transferase |
| gene_02800 | Transporter |  | dltA | D-alanine-D-alanyl carrier protein ligase |
| gene_02879 | Hypothetical protein |  | gene_06841 | non-ribosomal peptide synthetase/polyketide synthase |
| gene_02945 | Polyketide synthase |  | gene_06945 | Hypothetical protein |
| blsF | CGA synthase-related protein |  | gene_07037 | Hypothetical protein |
| gene_03059 | beta-ACP synthase |  | gene_07052 | Hypothetical protein |
| gene_03521 | Hypothetical protein |  | gene_07146 | Serine/threonine protein kinase |
| gene_03555 | cell division protein FtsK |  | gene_07163 | ABC transporter ATP-binding protein |
| gene_03563 | MFS transporter |  | gene_07200 | HAD family hydrolase |
| gene_03737 | Hypothetical protein |  | gene_07311 | Pyruvate, phosphate dikinase |
| hpt | Hypoxanthine phosphoribosyltransferase |  | gene_07326 | Bcr/CflA family drug resistance efflux transporter |
| gene_03814 | coenzyme F420 biosynthesis-associated protein |  | gene_07480 | IS5 family transposase |
| gene_03883 | non-ribosomal peptide synthetase |  | gene_07507 | Hypothetical protein |
| gene_03935 | Hypothetical protein |  | gene_07546 | Hypothetical protein |
| gene_03939 | Hypothetical protein |  | gene_07864 | Pyridine nucleotide-disulfide oxidoreductase |

**Table S10:** Genes containing non-synonymous mutations in the majority of *S. lydicus* 100-isolate pools at one site at 24 months but no mutations at any other site. No such mutations existed for *P. megaterium* populations at 24 months.

| **BC UNIQUE GENES** | |
| --- | --- |
| **Gene ID** | **Gene Annotation** |
| *gene_02629* | hypothetical protein |
|  | |
| **GH UNIQUE GENES** | |
| **Gene ID** | **Gene Annotation** |
| *clpB* | ATP-dependent chaperone ClpB |
| *gcl* | glyoxylate carboligase |
| *gene_00822* | phosphomethylpyrimidine synthase ThiC |
| *gene_03353* | dipeptide/oligopeptide/nickel ABC transporter ATP-binding protein |
| *gene_03363* | serine hydroxymethyltransferase |
| *gene_03756* | dihydroxy-acid dehydratase |
| *gene_07848* | aldehyde dehydrogenase |
| *katG_1* | catalase/peroxidase HPI |
| *tuf_3* | elongation factor Tu |

**SUPPLEMENTAL INFORMATION**

**METHODS**

*aChip construction details*

Empty plastic 1000-µL tip rack inserts (VWR International, LLC, Cat. No. 89079-470) were cut into rectangular pieces each containing 12 holes and were then autoclaved. All subsequent construction occurred inside a biosafety cabinet to maintain sterility. A Polycarbonate Track Etched (PCTE) membrane with a 30 nm pore size (GVS Filter Technology, Sandford, ME, USA) was cut with ethanol-sprayed scissors into pieces marginally larger than the plastic chips and affixed to one side of each aChip with silicone caulking glue (Loctite Re-New, Henkel Corporation, Rocky Hill, CT, USA) and allowed to dry (**Figure 1B**). The cure chemistry of Loctite Re-New is condensation curing. This sealant does contain two biocidal compounds: titanium dioxide and 2-(4-Thiazolyl)benzimidazole (https://www.buildsite.com/pdf/pl/Loctite-RE-NEW-Specialty-Silicone-Sealant-SDS-1868631.PDF). However, the mode of action of the former requires light to generate reactive oxygen species and would have been inactive underground, while the latter is primarily a fungicide, so neither should have strongly affected our focal bacterial species. Such properties should be considered when choosing a sealant for similar devices in future experiments.

The carrier soil was originally collected from a monoculture corn field at the Russell E. Larson Agricultural Research Station (40°43’19.4”N 77°55’43.0”W) in June 2019, and sieved at 2mm to homogenize and remove rocks and plant debris. The soil was then autoclaved for 60 minutes at 121ºC two times with a 24-h incubation between each round of autoclaving, to encourage any surviving microbes to break dormancy before the next round of autoclaving. The soil was autoclaved at its field moisture and was not re-wetted between the two autoclave runs. Although gamma irradiation is often used for soil sterilization, we have found it less reliable and more costly than multiple rounds of autoclaving, which is supported by other studies [1, 2], and subsequent microbial recolonization is very similar across both methods [3].

After the two rounds of autoclaving, we inoculated the carrier soil with our ancestral isolates (5mL of overnight TSB culture of *P. megaterium* OR 4-day old TSB culture of *S. lydicus* (unwashed) into 750mL of the twice-autoclaved carrier soil). We then incubated the inoculated carrier soil at 20°C for four weeks. At the time of aChip construction, the density of *P. megaterium* cells in the carrier soil as determined by dilution plating on TSA was 2.76 x 10^8^ colony forming units (CFU) g^-1^ of fresh soil, and the density of the *S. lydicus* cells as determined by dilution plating on ISP4 was 1.02 x 10^8^ CFU g^-1^ fresh soil. The carrier soil was then allocated into the wells of each half-constructed aChip, approximately 1g per aChip (**Figure 1B**). Silicone glue was then carefully spread and a second PCTE membrane was affixed, creating a complete seal around each well (**Figure 1B**).

*100-isolate pools*

To recapture *P. megaterium* or *S. lydicus* isolates from the retrieved aChips, 50 mg of carrier soil was thoroughly suspended and homogenized in 10 mL of sterilized water for dilution plating on TSA or ISP4, respectively. We then transferred 100 colonies with morphologies identical to that the appropriate ancestral isolate from the soil suspension dilution plates onto gridded, 100mm diameter TSA or ISP4 plates (one colony per grid cell). Generally this captured the first 100 colonies encountered on the dilution plates, starting with the most dilute plates. Once transferred, colonies on the gridded plates were incubated at 28 ºC for 24 h for *P. megaterium*, or 1 week for *S. lydicus*. The gridded plates were then flooded with 4 mL autoclaved distilled water and sterile single use cell spreaders used to suspend and mix the colonies or spores. This suspension, containing approximately equal amounts of each of the 100 isolates, was then pipetted into sterile plastic tubes and gently vortexed to homogenize. We used 1mL of suspension for metagenomic sequencing and the remainder was stored as glycerol stock at -80ºC.

*DNA extraction and sequencing*

For aChip carrier soil samples and *S. lydicus* spore suspensions, DNA was extracted using the NucleoSpin 96 Soil DNA extraction kit (Macherey-Nagel, Düren, Germany). For *P. megaterium* cell suspensions, DNA was extracted using the GenElute Bacterial Genomic DNA kit (Sigma-Aldrich Co., St. Louis, MO, USA). DNA extracts were prepared for sequencing with the Illumina DNA PCR-free prep kit (Illumina, San Diego, CA, USA) at the Penn State University Park Genomics Core Facility and sequenced 2x150bp on an Illumina NovaSeq at the Penn State Hershey Genome Science Facility.

*Bioinformatic analysis*

Read quality was assessed using FastQC v0.11.9 and default parameters [4]. Adapter sequences and low-quality bases were removed with Trimmomatic v0.36 using default parameters [5]. The ancestral genomes were assembled *de novo* from trimmed reads with SPAdes v3.15.0 [6], using the “careful” option and k-mer lengths of 99 and 127. Average coverage was calculated using BWA v0.7.17 [7] and SAMtools v1.12 [8], and assembly quality was checked with QUAST v5.1.0rc1 [9]. Select quality metrics of the ancestral *P. megaterium* and *S. lydicus* genomes used as a reference for later mutation analysis are displayed in **Table S3**. The assembled ancestral genomes were annotated with Prokka v1.14.6 [10] using default settings.

For metagenomic sequences from soil DNA, we used Kraken v2.1.2 to assign taxonomy to the trimmed sequencing reads [11] using paired reads as input. Kraken output excluding the focal species was transformed into an OTU-style table using a python script “kraken2OTU” (<https://github>.com/sipost1/kraken2OTUtable) and imported in R version 4.3.1 to examine non-focal species presence and diversity. Heatmaps were generated using R package *pheatmap.* We also used Kraken to double check the purity of our 100-isolate pools, which should have contained nearly 100% *P. megaterium* or *S. lydicus* reads. This was true for all *P. megaterium* 100-isolate pools, but not for the *S. lydicus* 3-month and 10-month 100-isolate pools (**Figure S3B**). This implies that there were contaminants present inside colonies that otherwise looked like *S. lydicus*, or that these 100-isolate DNA extracts were compromised at another point during sample preparation and sequencing (e.g. from the DNA extraction kit [12]). In either case, the resulting 100-isolate pool sequences generated significantly lower coverage of the *S. lydicus* genome (**Figure S3A**). As such, we considered these samples unusable.

For our mutation identification, we used breseq v0.35.5 (Deatherage and Barrick 2014) in polymorphism mode, with either our ancestral *P. megaterium* or *S. lydicus* genome as the reference and trimmed reads from the 100-isolate pools as the input. This program maps each individual sequencing read to the reference genome, and automatically discards reads that it cannot align well. There was near perfect agreement between breseq and Kraken on the degree of purity in our 100-isolate pools (**Figure S3B**), and we therefore relied on breseq to exclude non-focal species reads from the mutation analysis. Once reads are mapped, breseq identifies and annotates genetic variation in the aligned reads, and provides population-level frequency information for each identified mutation. Due to using short-read sequencing and ancestral genomes made up of multiple contigs, we had a limited ability to assess the presence of larger indels and structural variants and therefore focused on point mutations. We also used breseq for a coarse mutation analysis on the *P. megaterium* and *S. lydicus* pre-deployment carrier soil DNA, first using the extract_kraken_reads.py script from the KrakenTools suite (Lu et al 2022) to extract only those reads classified as *P. megaterium* or *S. lydicus*. Mutation lists generated by breseq were then imported into R v4.2.1 (R-Core-Team 2024) for analysis.

*Statistical analysis*

Two-way ANOVAs were used to test the effect of aChip burial site, harvest time, and the site*time interaction on some mutation frequencies for *P. megaterium* populations using R package *stats*. Partial eta-squared values with confidence intervals were calculated to quantify effect sizes from ANOVA results using R package *effectsize.* Post-hoc comparisons were made using Tukey’s HSD test. To explore non-focal species population patterns within the harvested aChips, ANOVA and Tukey’s HSD test were used to assess change in alpha diversity (Shannon index) across time while PERMANOVA (R packages *vegan* and *pairwiseAdonis*) was used to assess the impact of burial site and time on Bray-Curtis beta diversity. Before performing the PERMANOVA, we confirmed the assumption of homogeneity of group dispersions across burial site x harvest time treatments groups using function “betadisper” from R package *vegan*.

1. Lees, K., et al., *Soil sterilisation methods for use in OECD 106: How effective are they?* Chemosphere, 2018. **209**: p. 61-67.

2. McNamara, N., et al., *Effects of acute gamma irradiation on chemical, physical and biological properties of soils.* Applied Soil Ecology, 2003. **24**(2): p. 117-132.

3. King, W., L., et al., *Autoclaving is at least as effective as gamma irradiation for biotic clearing and intentional microbial recolonization of soil.* mSphere, 2024. **9**(7): p. e00476-24.

4. Trivedi, U.H., et al., *Quality control of next-generation sequencing data without a reference.* Frontiers in Genetics, 2014. **5**(111).

5. Bolger, A.M., M. Lohse, and B. Usadel, *Trimmomatic: a flexible trimmer for Illumina sequence data.* Bioinformatics, 2014. **30**(15): p. 2114-2120.

6. Bankevich, A., et al., *SPAdes: a new genome assembly algorithm and its applications to single-cell sequencing.* Journal of computational biology, 2012. **19**(5): p. 455-477.

7. Li, H. and R. Durbin, *Fast and accurate short read alignment with Burrows–Wheeler transform.* Bioinformatics, 2009. **25**(14): p. 1754-1760.

8. Li, H., et al., *The Sequence Alignment/Map format and SAMtools.* Bioinformatics, 2009. **25**(16): p. 2078-2079.

9. Gurevich, A., et al., *QUAST: quality assessment tool for genome assemblies.* Bioinformatics, 2013. **29**(8): p. 1072-1075.

10. Seemann, T., *Prokka: rapid prokaryotic genome annotation.* Bioinformatics, 2014. **30**(14): p. 2068-2069.

11. Wood, D.E., J. Lu, and B. Langmead, *Improved metagenomic analysis with Kraken 2.* Genome biology, 2019. **20**(1): p. 1-13.

12. Fierer, N., et al., *Guidelines for preventing and reporting contamination in low-biomass microbiome studies.* Nature Microbiology, 2025. **10**(7): p. 1570-1580.
