# Supplementary figures and images for: The adaptation chip: repurposing the principles of the ichip for guiding in situ experimental evolution
Source: ISME Commun. 2026 Apr 3;6(1):ycag053. doi: 10.1093/ismeco/ycag053 (PMC13064666; doi:10.1093/ismeco/ycag053)

Non-focal taxa in *P. megaterium* aChips

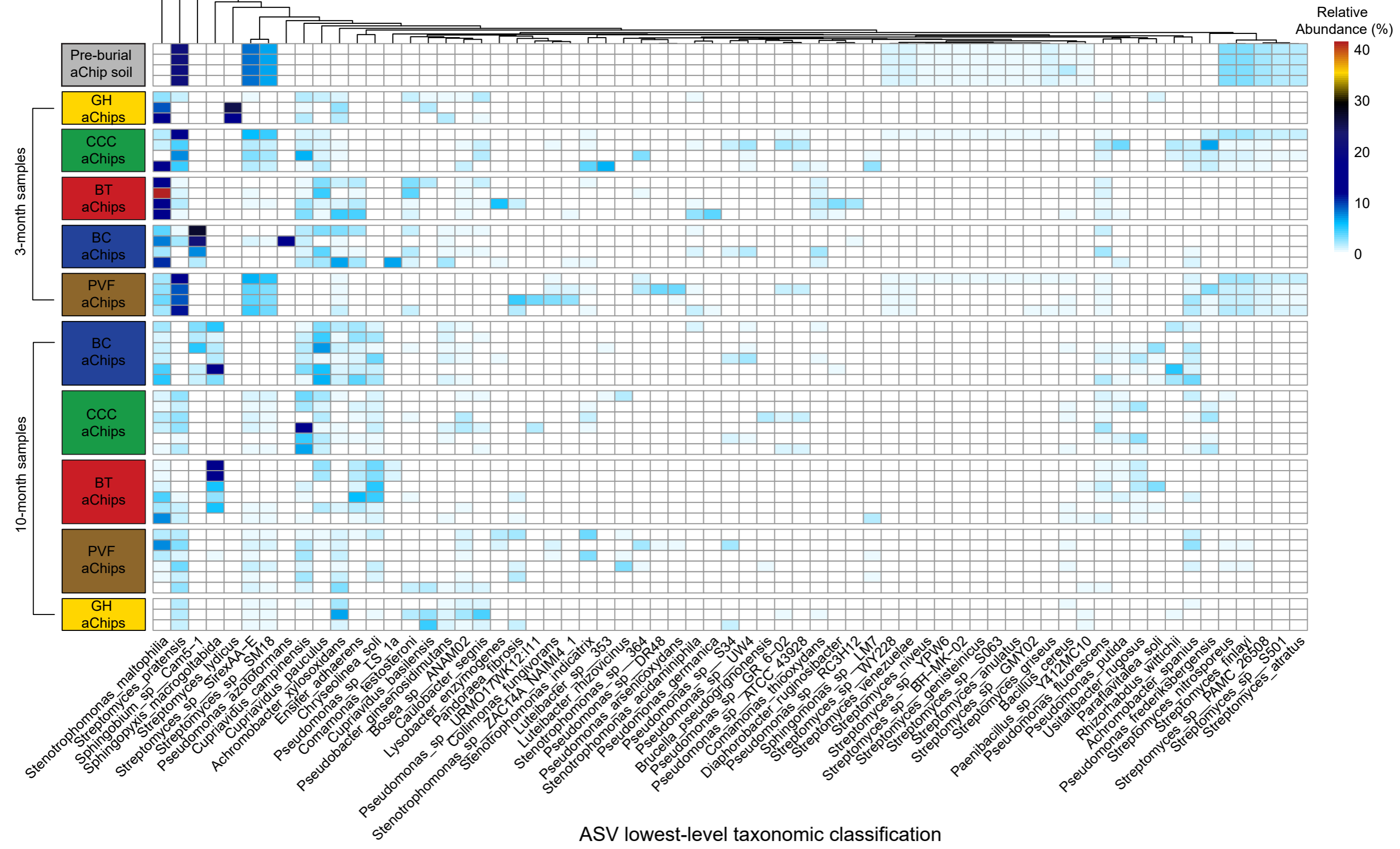

Supplement: Supplementary_materials_ycag053 [file supplementary_materials_ycag053.zip › FigureS4_Heatmap_Pmeg_nonfocal.pdf]

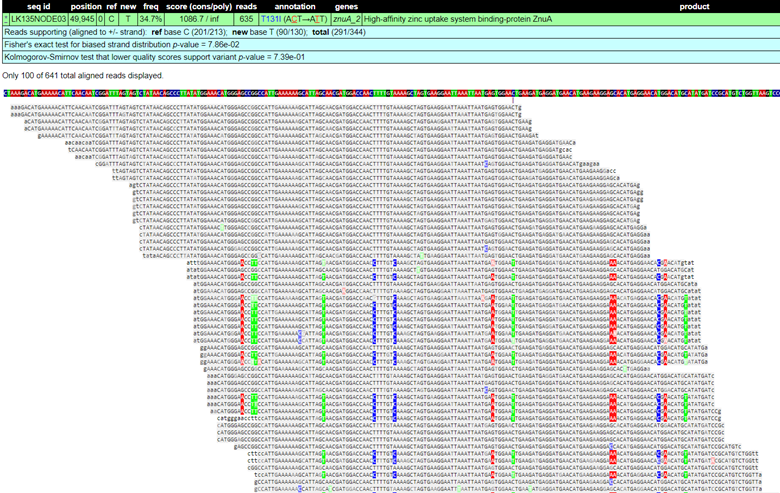

Supplement: Supplementary_materials_ycag053 [file supplementary_materials_ycag053.zip › FigureS6_breseq_mutation_screenshot.png]
